# Diversity of Influenza A(H5N1) Viruses in Infected Humans, Northern Vietnam, 2004–2010

**DOI:** 10.3201/eid2407.171441

**Published:** 2018-07

**Authors:** Hirotaka Imai, Jorge M. Dinis, Gongxun Zhong, Louise H. Moncla, Tiago J.S. Lopes, Ryan McBride, Andrew J. Thompson, Wenjie Peng, Mai thi Q. Le, Anthony Hanson, Michael Lauck, Yuko Sakai-Tagawa, Shinya Yamada, Julie Eggenberger, David H. O’Connor, Yasuo Suzuki, Masato Hatta, James C. Paulson, Gabriele Neumann, Thomas C. Friedrich, Yoshihiro Kawaoka

**Affiliations:** University of Wisconsin, Madison, Wisconsin, USA (H. Imai, J.M. Dinis, G. Zhong, L.H. Moncla, T.J.S. Lopes, A. Hanson, M. Lauck, J. Eggenberger, D.H. O’Connor, M. Hatta, G. Neumann, T.C. Friedrich, Y. Kawaoka);; The Scripps Research Institute, La Jolla, California, USA (R. McBride, A.J. Thompson, W. Peng, J.C. Paulson);; National Institute of Hygiene and Epidemiology, Hanoi, Vietnam (M.t.Q. Le);; University of Tokyo, Tokyo, Japan (Y. Sakai-Tagawa, S. Yamada, Y. Kawaoka);; Chubu University, Kasugai, Japan (Y. Suzuki)

**Keywords:** influenza virus, H5N1 subtype, genetic diversity, adaptation, influenza, viruses, humans, Vietnam, respiratory infections

## Abstract

Influenza viruses exist in each host as a collection of genetically diverse variants, which might enhance their adaptive potential. To assess the genetic and functional diversity of highly pathogenic avian influenza A(H5N1) viruses within infected humans, we used deep-sequencing methods to characterize samples obtained from infected patients in northern Vietnam during 2004–2010 on different days after infection, from different anatomic sites, or both. We detected changes in virus genes that affected receptor binding, polymerase activity, or interferon antagonism, suggesting that these factors could play roles in influenza virus adaptation to humans. However, the frequency of most of these mutations remained low in the samples tested, implying that they were not efficiently selected within these hosts. Our data suggest that adaptation of influenza A(H5N1) viruses is probably stepwise and depends on accumulating combinations of mutations that alter function while maintaining fitness.

Highly pathogenic avian influenza (HPAI) A(H5N1) viruses are not readily transmitted among humans, although a few cases of human-to-human transmission have been reported (*1*–*3*). However, recent laboratory experiments have demonstrated that a small number of amino acid changes can render avian H5 influenza viruses transmissible via respiratory droplets among ferrets (*4*–*6*) or guinea pigs (*7*). The evolutionary processes by which influenza A(H5N1) viruses might adapt to mammals are poorly understood. Because of error-prone genome replication, influenza viruses exist in an infected host as a collection of genetic variants; this within-host genetic diversity is believed to facilitate rapid adaptation to changing selective pressures (*8*). Recent mathematical models have highlighted the role of virus mutation rates, the number of replication cycles in a given host, and natural selection in assessing the likelihood with which an influenza A(H5N1) virus transmissible among mammals might emerge, but little information on avian influenza virus diversity within infected hosts was available to inform these studies (*9*,*10*). 

We recently found that selection pressure on hemagglutinin (HA) can impose a strong population bottleneck during virus transmission: a variant representing only 5.9% of the total virus population in animals infected with a reassortant influenza A(H5N1) virus infected an animal via aerosol transmission (*11*). These findings prompted us to seek a deeper understanding of virus populations in infected humans. We used deep-sequencing and functional analyses to evaluate the genetic and functional diversity among influenza A(H5N1) viruses in infected humans.

## Materials and Methods

### Biosafety and Biosecurity

The studies were conducted after the University of Wisconsin–Madison Office of Biologic Safety completed risk assessments for the proposed experiments and the Institutional Biosafety Committee approved the experiments. All experiments used the biosafety and biosecurity practices and procedures that were developed in conjunction with the University’s Select Agent Program and approved by the Centers for Disease Control and Prevention Division of Select Agents and Toxins and by the US Department of Agriculture, Animal and Plant Health Inspection Service, Agriculture Select Agent Services. The studies and subsequent data were reviewed for potential dual use research of concern in accordance with the US Government Policy for Oversight of Life Sciences Dual Use Research of Concern. All experiments conducted in laboratories at the University of Tokyo (Tokyo, Japan) were approved by the appropriate committees at the University of Tokyo. Isolation and amplification of HPAI A(H5N1) viruses from clinical specimens were performed in enhanced Biosafety Level 3 laboratories at the University of Tokyo, which are approved for such use by the Ministry of Agriculture, Forestry, and Fisheries, Japan.

### RNA Extraction, cDNA Synthesis, and PCR Amplification

We obtained clinical specimens (throat swabs and tracheal aspirates) from 7 patients infected with influenza A(H5N1) virus in northern Vietnam during 2004–2010. To avoid the emergence of mutations during virus amplification in cultured cells, we extracted total RNA directly from clinical specimens by using the QIAamp MinElute Virus Spin Kit (QIAGEN, Hilden, Germany). Because of the limited amounts of viral RNA (vRNA), we reverse transcribed vRNA segments encoding known determinants of mammalian adaptation or transmissibility (i.e., polymerase basic [PB] 2, PB1, polymerase acidic [PA], nucleoprotein [NP], HA, and nonstructural [NS] segments) by using SuperScript III (Invitrogen, Carlsbad, CA, USA). We PCR amplified the segments by using iProof High-Fidelity DNA polymerase (Bio-Rad, Hercules, California, USA) with primers specific for the terminal sequences of each vRNA segment. If we failed to PCR amplify the full-length vRNAs, we used primers specific for the internal sequences (Technical Appendix Table 1).

### Illumina MiSeq Sequencing

We quantified PCR-amplified and agarose gel–purified PCR products by using the Qubit dsDNA High Sensitivity Assay Kit (Invitrogen). Amplified genes were pooled, and sample barcodes were added by using the Nextera XT DNA Sample Preparation Kit (Illumina, San Diego, CA, USA). Barcoded samples were pooled in equimolar amounts and loaded onto a 500-cycle kit for sequencing on the Illumina MiSeq. Illumina MiSeq fastq files are publicly available in the Sequence Read Archive (https://www.ncbi.nlm.nih.gov/sra; SRA: SRP103022).

### Computational Methods

We imported Illumina MiSeq sequences into CLC Genomics Workbench version 7.3 (CLC bio, Aarhus, Denmark) in fastq format. We trimmed reads by using a quality-limit threshold of 0.001 and retained only reads >100 bp. Trimmed reads were de novo assembled to generate a consensus sequence for each gene segment of each patient sample. We then used these within-host consensus sequences, defined by the nucleotides found in more than half of the sequence reads at each position, as reference sequences for further mapping. Reads were remapped to each sample’s own consensus sequence to identify within-host single-nucleotide polymorphisms (SNPs), requiring that nucleotide positions be covered by >100 sequence reads, that they have a central base quality score >Q30, and that SNPs be detected in at least 1 forward and 1 reverse read. We only considered SNPs present in >1% of sequence reads within a population; we previously showed that this stringent cutoff excludes false-positive SNPs generated by using our approach (*11*). Amino acid variations in HA were mapped onto the A/Vietnam/1203/2004 (H5N1) HA structure (Protein Data Bank accession no. 2FK0) by using Pymol (http://www.pymol.org).

### Cells

MDCK cells (ATCC, Manassas, VA, USA) were maintained in minimal essential medium with 5% newborn calf serum. Human embryonic kidney 293T cells (ATCC) were maintained in Dulbecco modified Eagle medium with 10% fetal calf serum. Both cell lines were cultured at 37°C in 5% CO_2_.

### Viruses

Reassortants possessing an HA polymorphism in the genetic background of the respective H5 HA gene and the remaining genes derived from A/California/04/2009 (H1N1) (CA04) were generated in 293T cells by using reverse genetics (*12*) and amplified in MDCK cells; the resulting viruses are referred to as in the following example: CA04/UT3040I-HA-138V. Sanger sequence analysis of all virus stocks revealed that the CA04/UT36282I/II-HA-138V virus possessed a mixture of valine and alanine at HA position 138 and that the HA-203P residue of CA04/UT31312III-HA-203P was replaced by leucine. We were unable to rescue the CA04/UT31413II-HA-486H reassortant virus and therefore omitted it from further studies. Because of the research pause imposed on certain gain-of-function studies (http://www.phe.gov/s3/dualuse/Documents/gain-of-function.pdf), we were not able to generate 1 variant (CA04/UT31413II-HA-511I) and therefore will not discuss it further. All experiments with infectious viruses possessing the H5 HA with a polybasic cleavage site were performed in enhanced Biosafety Level 3 containment laboratories.

### Solid-Phase Binding Assay

We coated 96-well plates with sodium salts of sialylglycopolymers (poly-l-glutamic acid backbones containing N-acetylneuraminic acid linked to galactose through either an α2,3 [Neu5Acα2,3Galβ1,4GlcNAcβ1-Pap] or an α2,6 [Neu5Acα2,6Galβ1,4GlcNAcβ1-Pap] bond) (*13*), synthesized at Chubu University. We assessed virus binding to the sialylglycopolymers as previously described (*4*,*14*).

### Glycan Arrays

We performed glycan array analysis as previously described (*15*,*16*). Viruses were amplified in MDCK cells and inactivated by mixing the supernatants with 0.1% β-propiolactone (final concentration). A complete list of glycans on the array is provided in online Technical Appendix Figure 1.

**Figure 1:**
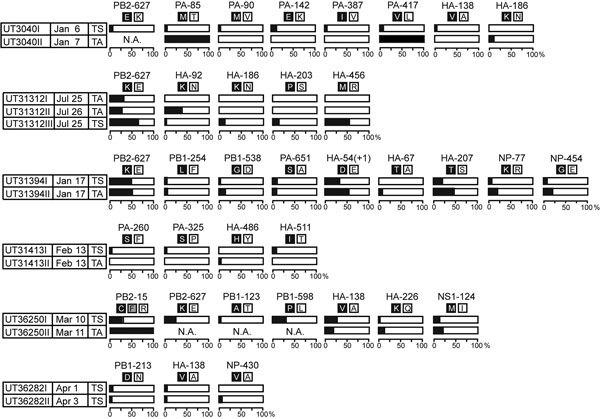
Frequencies of nonsynonymous single-nucleotide polymorphisms detected in >5% of sequence reads obtained from humans infected with influenza A(H5N1) virus, northern Vietnam, 2004–2010. Bar graphs depict the percentages of the indicated single-nucleotide polymorphisms from TS or TA samples. The amino acids at the respective positions are shown by the single-letter code. HA-54(+1) refers to an H5 HA amino acid insertion after position 54 that was not found in H3 HAs (which was used as a reference for the numbering of HA amino acid positions). HA, hemagglutinin; NP, nucleocapsid; NS, nonstructural; PA, polymerase acidic; PB, polymerase basic; TA, tracheal aspirate; TS, throat swab.

### Virus Tissue Binding Assay

We diluted inactivated viruses with phosphate-buffered saline to equal amounts of viral M1 protein (as assessed by Western blot). Diluted viruses were labeled with FITC isomer I (Life Technologies, Carlsbad, CA, USA). Virus binding to human adult normal trachea sections was assessed as previously described (*17*,*18*).

### Thermostability Assay

We diluted amounts of virus equivalent to 64 hemagglutinating units in minimal essential medium supplemented with 0.3% bovine serum albumin and heat treated the diluted virus samples at 55°C for the indicated times. We then determined the hemagglutination activity of the heat-treated viruses by using HA assays with 0.5% turkey red blood cells (Rockland Immunochemicals Inc., Limerick, PA, USA). Virus infectivity was determined by performing plaque assays in MDCK cells.

### Mini-Replicon Assay

We transfected human 293T cells with pCAGGS plasmids (*19*) encoding wild-type or mutant PB2, ​PB1, ​PA, and ​NP protein with a plasmid expressing the firefly luciferase gene from a virus-like RNA (pPolWSNNA F-Luc) (*20*) and with the control plasmid pGL4.74[hRluc/TK] (expressing *Renilla* luciferase; Promega, Madison, WI, USA) by using TransIT293 (Mirus, Madison, WI, USA). Cells were incubated at 33°C or 37°C for 24 h and then lysed with 1× Passive Lysis Buffer (Promega). We determined luciferase activity by using the Dual-Luciferase Reporter Assay System (Promega).

### Interferon Reporter Assays

We assessed the effect of NS1 amino acid changes on interferon production as previously described (*21*,*22*) with some modifications. We transfected human 293T cells with pCAGGS plasmids expressing wild-type or mutant NS1 and with a plasmid expressing luciferase under the control of the interferon-β promoter (pGL-interferonβ luc) (*23*). Luciferase activity was determined 24 h after treating cells with 10^6^ focus-forming units of Sendai virus to induce interferon production (*21*,*22*).

To assess the effect of NS1 on interferon signaling, we transfected 293T cells with a plasmid encoding wild-type or mutant NS1 and a plasmid encoding luciferase under the control of an interferon-stimulated response element (pISRE-Luc; Clontech, Kusatsu, Japan). Luciferase activity was determined after treating cells with human interferon-β1a (PBL Assay Science, Piscataway, NJ, USA).

### Statistical Analyses

For the mini-replicon assay, we used the Dunnett test to compare the viral polymerase activity of each mutant with that of the respective majority variant in each experiment. In the interferon reporter assays, we analyzed the data by using a one-way analysis of variance, followed by the Tukey post hoc test to compare the interferon antagonistic properties among the empty vector, UT36250I-NS1, and UT36250I-NS1-124M.

## Results

### Genetic Diversity of Influenza A(H5N1) Viruses in Humans

For each of the 7 patients with confirmed influenza A(H5N1) virus infection, 2–3 samples were available that had been collected on different days or from different anatomic sites (i.e., throat swabs or tracheal aspirates) (Table). To estimate the genetic diversity of these viruses in humans, we first defined a within-host consensus sequence for each viral gene segment, the sequences of which we obtained by deep sequencing with RNA extracted from patient clinical samples. We then counted the SNPs relative to this consensus that were detected at >1% frequency within each host, detecting a total of 251 nonsynonymous and 146 synonymous mutations (Technical Appendix Table 2). Within-host genetic diversity varied considerably among patients, anatomic site of sample (nasal swab or tracheal aspirate), and day of isolation (Technical Appendix Figure 2, Table 3). Nonetheless, 91 variants were detected >2 times in samples from the same patient.

**Table:** Characteristics of patients infected with influenza A(H5N1) virus and samples collected, northern Vietnam, 2004–2010*

Patient no., virus isolated	HA clade	Sample	Date			Outcome
			Symptom onset	Hospitalization	Sample collection	
UT3040						
A/Vietnam/UT3040I/2004 (UT3040I)	1	TS	NA	NA	2004 Jan 6	Died
A/Vietnam/UT3040II/2004 (UT3040II)	1	TA	NA	NA	2004 Jan 7	Died
UT31312						
A/Vietnam/UT31312I/2007 (UT31312I)	2.3.4	TA	NA	NA	2007 Jul 25	Died
A/Vietnam/UT31312II/2007 (UT31312II)	2.3.4	TA	NA	NA	2007 Jul 26	Died
A/Vietnam/UT31312III/2007 (UT31312III)	2.3.4	TS	NA	NA	2007 Jul 25	Died
UT31394						
A/Vietnam/UT31394I/2008 (UT31394I)	2.3.4	TS	NA	NA	2008 Jan 17	Died
A/Vietnam/UT31394II/2008 (UT31394II)	2.3.4	TA	NA	NA	2008 Jan 17	Died
UT31413						
A/Vietnam/UT31413I/2008 (UT31413I)	2.3.4	TS	2008 Feb 3	NA	2008 Feb 13	Died
A/Vietnam/UT31413II/2008 (UT31413II)	2.3.4	TA	2008 Feb 3	NA	2008 Feb 13	Died
UT36250						
A/Vietnam/UT36250I/2010 (UT36250I)	2.3.4.2	TS	2010 Mar 5	2010 Mar 10	2010 Mar 10	Survived
A/Vietnam/UT36250II/2010 (UT36250II)	2.3.4.2	TA	2010 Mar 5	2010 Mar 10	2010 Mar 11	Survived
UT36282						
A/Vietnam/UT36282I/2010 (UT36282I)	2.3.4.1	TS	2010 Mar 27	2010 Apr 2	2010 Apr 1	Survived
A/Vietnam/UT36282II/2010 (UT36282II)	2.3.4.1	TS	2010 Mar 27	2010 Apr 2	2010 Apr 3	Survived
UT36285						
A/Vietnam/UT36285I/2010 (UT36285I)	2.3.4.1	TS	2010 Apr 2	2010 Apr 4	2010 Apr 4	Survived
A/Vietnam/UT36285II/2010 (UT36285II)	2.3.4.1	TS	2010 Apr 2	2010 Apr 4	2010 Apr 8	Survived

*HA, hemagglutinin; NA; not available; TA, throat swab; TS, tracheal aspirate.

Previously, we demonstrated that a viral variant detected at a frequency of only 5.9% in 1 ferret founded infection after respiratory droplet transmission to a contact ferret (*11*). Therefore, we focused our functional analyses on mutants present in >5% of the virus population, yielding 29 nonsynonymous polymorphisms in the viral HA, PB2, PB1, PA, NP, and NS1 proteins of viruses obtained from 6 patients (Figure 1; Technical Appendix Table 3). One more variant (PA-85T/M) was detected at a frequency of <5% at the first time point, but its frequency had increased greatly by the second time point; therefore, we included this variant in our subsequent analyses, for a total of 30 variants examined (Figure 1; Technical Appendix Table 3).

### Receptor-binding Specificity of HA Variants

**Figure 2:**
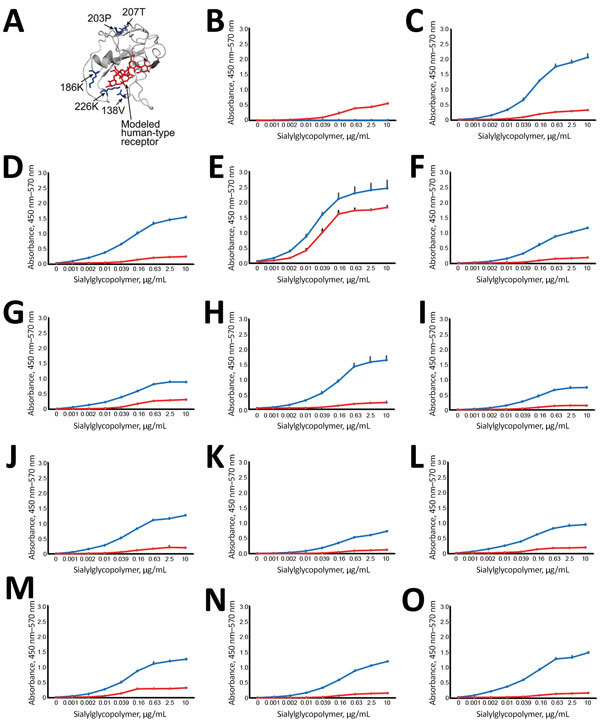
Effect of amino acid variations in hemagglutinin (HA) on influenza virus receptor-binding specificities in influenza A(H5N1) virus isolates from humans, northern Vietnam, 2004–2010. A) Localization of selected amino acid variations detected in clinical samples. The detected HA changes were mapped onto the receptor-binding domain (aa positions 117–265) of a monomer of A/VN1203/2004 (H5N1) HA (Protein Data Bank accession no. 2FK0). Red indicates modeled human-type receptor; blue indicates positions of amino acid variations on the receptor-binding domain. B–O) Receptor-binding specificities of HA variants. Direct binding of virus to α2,3-linked (blue) or α2,6-linked (red) sialylglycopolymers was assessed. Shown is the mean receptor-binding specificity + SDs of triplicates of a single experiment. If 2 isolate numbers are listed (e.g., CA04/UT3040I/II), we tested the major sequence variant (which is identical between the 2 samples: B) CA04/K173; C) CA04/UT3040I/II-HA; D) CA04/UT3040I-HA-138V; E) CA04/UT3040II-HA-186K; F) CA04/UT31312III-HA; G) CA04/UT31312III-HA-186K; H) CA04/UT31312III-HA-203P-to-L (the proline residue introduced at position 203 of UT31312III HA mutated to leucine); I) CA04/UT31394II-HA; J) CA04/UT31394II-HA-207T; K) CA04/UT36250I/II-HA; L) CA04/UT36250I/II-HA-138V; M) CA04/UT36250II-HA-226K; N) CA04/UT36282I/II-HA; O) CA04/UT36282I/II-HA-138V/A (after introduction of valine at position 138 of UT36282I/II HA, we detected both valine and alanine at this position).

**Figure 3:**
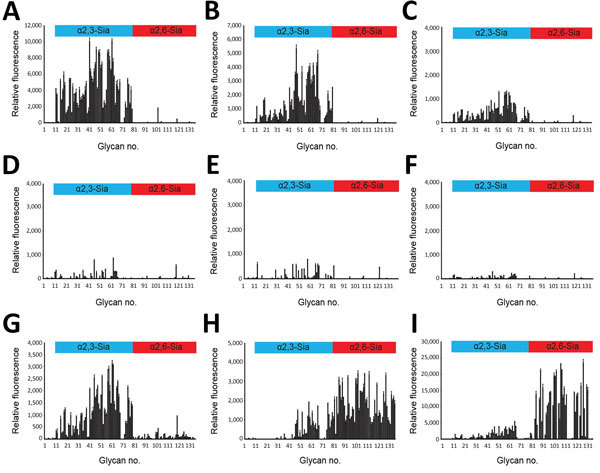
Glycan microarray analysis of selected influenza A(H5N1) virus isolates from humans, northern Vietnam, 2004–2010. The labeled viruses were applied to a microarray that included α2,3-linked (blue) and α2,6-linked (red) glycans, which are indicated by numbers on the *x*-axis (Technical Appendix Figure 1). Shown are the binding signals with error bars reflecting SEMs calculated from 4 of 6 replicates on the array after discarding the highest and lowest signals; note that the scales on the y-axis (relative fluorescence) vary because of differences in binding strength: A) CA04/UT3040I/II-HA; B) CA04/UT3040I-HA-138V; C) CA04/UT3040II-HA-186K; D) CA04/UT36250I/II-HA; E) CA04/UT36250I/II-HA-138V; F) CA04/UT36282I/II-HA; G) CA04/UT36282I/II-HA-138V/A; H) CA04/K173; I) Brisbane/10/2007.

Several of the HA sequence polymorphisms detected were located in the vicinity of the receptor-binding pocket (Figure 2, panel A). We therefore tested the receptor-binding properties of these variants by using a solid-phase binding assay with synthetic α2,3- or α2,6-linked sialylglycopolymers (Figure 2, panels B–O). A virus possessing the HA gene from the human A/Kawasaki/173/2001 (H1N1) virus and the remaining genes from CA04 (CA04/K173) served as a control virus for human virus receptor-binding specificity. As expected, this virus preferentially bound to α2,6-linked sialylglycopolymers (Figure 2, panel B). In contrast, CA04-reassortant H5 viruses encoding the majority and minority variants from infected persons preferentially bound to avian-type α2,3-linked sialylglycopolymers (Figure 2, panels C–O). In the context of CA04/UT3040II-HA, the HA-186K variant bound more efficiently to human-type receptors than did consensus CA04/UT3040II (encoding HA-186N) (Figure 2, panels C and E); however, this effect was not detected with the same viruses in the glycan arrays (Figure 3, panels A, C), or when we tested the same mutation in UT31312III HA (Figure 2, panels F, G). Therefore, more rigorous evaluation is needed to assess the role of 186K in human-type receptor specificity.

We also performed a glycan array analysis on an array containing 135 synthetic glycans representing O-linked and N-linked glycans and linear glycan fragments with sialic acid in α2,3- and α2,6-linkages (Technical Appendix Figure 1) (*15*). Glycan arrays can be used to evaluate the specificity of viruses for numerous glycans simultaneously and to assess avian- and human-type specificity with high stringency; however, they are less effective for assessing changes in avidity (*24*). Tested wild-type and mutant influenza A(H5N1) viruses interacted primarily with α2,3-linked glycans (Figure 3, panels A–G); however, some mutations affected the binding intensity to receptor glycans (e.g., compare Figure 3, panels F and G). Two human control viruses (CA04/K173 and A/Brisbane/10/2007) that we used in previous assays bound primarily to α2,6-linked glycans (Figure 3, panels H, I). Collectively, these data demonstrate that the H5 HA variants detected in infected humans retained their preferential binding to avian-type receptors.

### Binding of HA Variants to Human Respiratory Tissues

We also tested the HA variants for their ability to bind to human respiratory airway samples (Technical Appendix Figure 3). The human CA04/K173 virus bound extensively to epithelial cells of tracheal tissue cross-sections. The CA04/UT3040I/II-HA virus bound weakly to mucus secreted by goblet cells but not to epithelial cells. Unlike the results of our solid-phase binding assays, CA04/UT36250I/II-HA-138V, CA04/UT36282I/II-HA, and CA04/UT36282I/II-HA-138V/A displayed appreciable binding to epithelial cells in human tracheal tissues (Technical Appendix Figure 3). To confirm virus binding specificity to sialic acids, we pretreated the tracheal tissues with *Arthrobacter ureafaciens* neuraminidase, which removes terminal sialic acids; as expected, virus binding to these samples was greatly reduced (Technical Appendix Figure 4). The remaining viruses displayed little to no binding to tracheal epithelial cells. These findings show that although we detected polymorphisms at HA amino acid positions known to affect receptor binding, the variants tested did not acquire preferential binding to human-type receptors, although the HA-138V mutation in UT36250I and the HA-138V/A mutation in UT36282I/II enhanced HA binding to epithelial cells in human respiratory tissue.

### Stability of HA Variants

**Figure 4:**
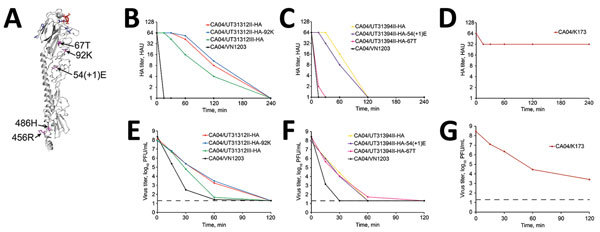
Effect of amino acid variations in HA on virus thermostability in influenza A(H5N1) virus isolates from humans, northern Vietnam, 2004–2010. A) Amino acid substitutions in non–receptor-binding domains mapped on the 3-dimensional structure of the monomer of VN1203 HA (Protein Data Bank accession no. 2FK0). Red indicates modeled human-type receptor; purple indicates positions of amino acid variations on the non–receptor-binding domains; blue indicates positions of amino acid variations on the receptor-binding domain corresponding to Figure 2, panel A. B–G) Thermostability of HA variants depicted in panel A. Amounts of viruses equivalent to 64 HA units were incubated at 55°C for 15, 30, 60, 120, and 240 min. B–D) HA titers in heat-treated samples were determined by performing HA assays with 0.5% turkey red blood cells: B) CA04/UT31312II-HA, CA04/UT31312II-HA-92K, CA04/UT31312III-HA, CA04/VN1203; C) CA04/UT31394II-HA, CA04/UT31394II-HA-54(+1)E, CA04/UT31394II-HA-67T, CA04/VN1203; D) CA04/K173. E–G) Virus titers of heat-treated samples determined by means of plaque assays in MDCK cells. Shown are the mean HA or virus titers of triplicates from a single experiment. Dashed lines indicate the detection limit for virus titration (20 PFU/mL). E) CA04/UT31312II-HA, CA04/UT31312II-HA-92K, CA04/UT31312III-HA, CA04/VN1203; F) CA04/UT31394II-HA, CA04/UT31394II-HA-54(+1)E, CA04/UT31394II-HA-67T, CA04/VN1203; G) CA04/K173. CA04/K173 virus was used as a control. HA, hemagglutinin.

We (*4*) and others (*25*) have shown that increased HA stability is needed for the respiratory droplet transmissibility of H5 viruses among ferrets. We therefore tested the HA polymorphisms that were located away from the receptor-binding domain (Figure 4, panel A) for their effect on HA thermostability (Figure 4, panels B–G). The selected variants were incubated at 55°C for the indicated time intervals, after which loss of infectivity was determined by means of a plaque assay and HA hemagglutination activity by hemagglutination assay.

The human CA04/K173 virus displayed appreciably higher thermostability than the avian control virus CA04/VN1203 (possessing the A/Vietnam/1203/2004 [H5N1] HA gene in the genetic background of CA04). No appreciable differences in thermostability were detected between the minority and majority variants in CA04/UT31312II/III (Figure 4, panels B, E) and in CA04/UT31394I/II (Figure 4, panels C, F) except that CA04/UT31394II-HA-67T lost hemagglutination activity more rapidly than the other variants for unknown reasons. We did not, therefore, identify HA-stabilizing mutations that arose during replication in humans.

### Polymerase Activity of Polymerase and NP Variants

**Figure 5:**
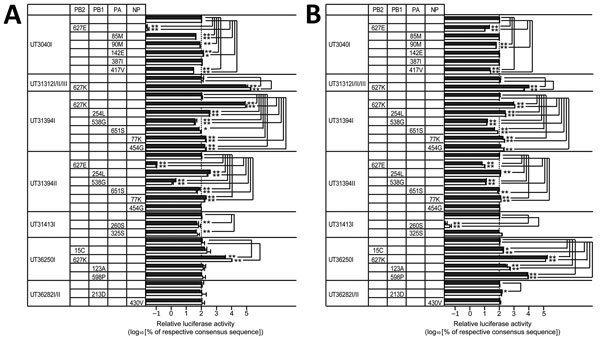
Effect of amino acid variations in polymerases and NP on viral polymerase activity in influenza A(H5N1) virus isolates from humans, northern Vietnam, 2004–2010. 293T cells were transfected with plasmids encoding the viral replication complex (PB2, PB1, PA, and NP), with a plasmid for the expression of an influenza virus mini-genome that encodes the firefly luciferase gene, and with a plasmid encoding *Renilla* luciferase (transfection control). If 2 or 3 isolate numbers are listed, we tested the major sequence variant, which is identical among the samples. The cells were incubated at 33°C (A) or at 37°C (B) for 24 h, and firefly and *Renilla* luciferase activities were measured by use of the Dual-Luciferase Reporter Assay System (Promega, Madison, WI, USA). The firefly luciferase values were divided by the *Renilla* luciferase values to normalize for variances in transfection efficiency. The experiments (each in triplicate) were independently repeated twice. The mean relative viral polymerase activities plus SDs of each independent experiment are shown as black and white bars. The viral polymerase activity of the respective majority variant was set to 100%. NP, nucleocapsid; PA, polymerase acidic; PB, polymerase basic; *p<0.05; **p<0.01 (both by Dunnett test).

Amino acid variations were located in multiple domains of each polymerase subunit. Our mini-replicon assay demonstrated that PB2-627K conferred significantly higher polymerase activity in mammalian cells than did PB2-627E (Figure 5), as demonstrated previously by us (*26*) and others (*25*). In addition, the UT36250I PB1-598P minority variant showed an ≈80-fold increase in polymerase activity at 37°C, but not at 33°C, relative to the PB1-598L majority variant (Figure 5, panels A and B). These potentially advantageous mutations did not become dominant in some of the samples available for this study; however, we cannot know whether these mutations became dominant at later times for which samples are not available. Two mutations (PA-85M and PA-417V) occurred at a low frequency in UT3040I yet represented the only population in UT3040II. Of note, both mutations reduced polymerase activity at 1 or both temperatures tested (Figure 5).

In summary, we detected a complex pattern of phenotypic effects of avian virus polymerase complex mutations in infected humans. With the exception of PB2-627K, some mutations improved polymerase activity in mini-replicon assays but did not become dominant in the virus samples tested, whereas other mutations replaced the wild-type amino acid although they appeared to reduce polymerase activity in vitro. Thus, enhancement of polymerase activity in the mini-replicon assay did not necessarily correlate with mutations that became dominant in vivo.

### Interferon-antagonistic Activity of NS1 Variants

The influenza A virus NS gene encodes 2 proteins, 1 of which (NS1) counteracts host innate interferon responses (*27*). HPAI A(H5N1) viruses are relatively resistant to the antiviral effects of host interferon responses (*28*), which might, in part, result from specific point mutations in the NS1 proteins of some of these viruses (*29*). We detected only 1 nonsynonymous polymorphism in NS1, a methionine (minority variant) or isoleucine (majority variant) at position 124 of NS1 in samples UT36250I and UT36250II (Figure 1); the frequency of the NS1-124M minority variant increased slightly in samples collected on consecutive days. We compared the ability of both NS1 variants to interfere with interferon production and signaling. The UT36250I-NS1 (encoding the NS1-124I majority variant) and UT36250I-NS1-124M proteins showed similar ability to antagonize interferon production (Technical Appendix Figure 5, panel A). However, UT36250I-NS1-124M was slightly more efficient than UT36250I-NS1 at suppressing interferon signaling (Technical Appendix Figure 5, panel B). This finding might explain the slightly increased frequency of NS1-124M on the second day of sampling, although such slight differences in variant frequency and interferon signaling suppression might not be biologically significant.

## Discussion

Worldwide, HPAI A(H5N1) viruses have infected ≈850 persons but have yet to adapt to humans. Because viral genetic and phenotypic diversity might facilitate adaptation in infected persons, we performed deep-sequencing and functional assays for influenza A(H5N1) viruses isolated from humans. We had access to only throat swab and tracheal aspirate samples; virus populations in other anatomic sites, such as alveoli, might differ. Nonetheless, the virus populations we observed were diverse, but most variants were detected only transiently, at low frequencies, or both, including variants with potentially beneficial traits (e.g., the PB1-598P mutation).

We found only a few sequence variants in >1 patient; they included PB2-627E/K, HA-138A/V, and HA-186N/K. PB2-627K is a known determinant of mammalian adaptation that increases the replicative ability of avian influenza virus polymerase complexes in mammals (*26**, **30**,**31*). Despite its strong effect on adaptation in mammals, in some samples, it coexisted with PB2–627E (Figure 1). Similarly, HA-138V and HA-186K, which can increase binding to human-type receptors (*14*,*32*–*34*), were detected in 3 and 2 patient samples, respectively. Although the HA-186K mutation exhibited increased binding to human-type receptors for 1 strain in the sialylglycopolymer binding assay, it did not alter the overall avian-type receptor specificity of the variants assessed by the glycan arrays, and both HA-138V and HA-186K coexisted with other viral variants in the samples tested. All variants bound to a wide variety of avian-type receptors, with no clear differences in specificity. Besides PB2-627K, the PB1-598P minority variant conferred a sizable increase in polymerase activity when compared with the majority population (encoding PB1-598L) (Figures 1, 5). Position 598 of PB1 lies in the so-called thumb domain of PB1 (*35*), and the amino acid at this position might interact with the PA polymerase subunit. We do not know how the L-to-P change would affect influenza virus replication; mechanistic follow-up studies will be necessary to answer this question. There was no clear association between particular virus mutations and clinical outcomes of patients in this study. Indeed, even the PB2-627K mutation was not always detected in patients who died (e.g., patient UT31413).

The evolutionary forces that govern mammalian adaptation and the emergence of viruses capable of transmission among humans are largely unknown. RNA virus replication generates within-host genetic diversity that can rapidly change because of selective pressures (*36*,*37*). One might assume that in infected humans, positive selection favors the rapid outgrowth of variants that possess mammalian-type traits and that variants with human-adapting mutations might commonly be found as subpopulations within infected persons even if they were not present in most viruses. However, we detected few variants with such traits; only the known mammalian-adapting PB2-627K substitution became dominant in several virus populations. These findings might imply that for a variant to become dominant, additional potentiating mutation(s) are necessary within the same genetic background as the adapting mutation. In other words, adaptive mutations might alter virus phenotypes but might require the presence of potentiating mutations to maintain viral fitness. For instance, we (*4*) and others (*25*) have shown the crucial role of another phenotypic trait, increased HA stability, for the respiratory droplet transmissibility of H5 viruses among mammals. Thus, avian influenza virus adaptation to humans probably does not occur in a steady linear fashion; rather, it probably depends on the stepwise accumulation of potentiating mutations that favor the emergence of a particular adaptive mutation, followed by the accumulation of additional potentiating mutations that favor further adaptive mutations, and so on. Our study provides a framework for testing this hypothesis by using deep sequencing to analyze avian influenza virus populations within humans, followed by phenotypic characterization in the laboratory.

Technical Appendix Additional methods and results for study of diversity of influenza A(H5N1) viruses in infected humans, northern Vietnam, 2004–2010.
