# Supplementary material for: Diversity of Influenza A(H5N1) Viruses in Infected Humans, Northern Vietnam, 2004–2010
Source: Emerg Infect Dis. 2018 Jul;24(7):1128–238. doi: 10.3201/eid2407.171441 (PMC6038741; doi:10.3201/eid2407.171441)
Supplement: Technical Appendix — Additional methods and results for study of diversity of influenza A(H5N1) viruses in infected humans, northern Vietnam, 2004–2010. [file 17-1441-Techapp-s1.pdf]

Article DOI: https://doi.org/10.3201/eid2407.171441

Diversity of Influenza A (H5N1) Viruses in Infected Humans

Technical Appendix

Technical Appendix Table 1. Primer sequences used in this study

vRNA segment	Nucleotide positions of RT-PCR products*	Forward primer		Reverse primer	
		Name	Sequence	Name	Sequence
PB2	1–1784	PB2–1-24F	AGCGAAAGCAGGTCAAATATATTC	PB2–1762–1784R	TTGGGTACCAAGGATTGGAACGG
	532–2341	PB2–532–558F	CCAAATGAAGTGGGAGCTAGAATATTG	PB2–2318–2341R	AGTAGAAACAAGGTCGTTTTTAAA
	167–2277	PB2–167–186F	TGAAATGGATGATGGCAATG	PB2–2258–2277R	CGCTGTCTGGCTGTCAGTAA
PB1	1–1829	PB1–1-18F	AGCGAAAGCAGGCAAACC	PB1–1804–1829R	CGGATATTGTATAGATTTGGTCCTCC
	501–2341	PB1–501–522F	ATCGGGACGGCTAATAGATTTC	PB1–2317–2341R	AGTAGAAACAAGGCATTTTTTCACG
	106–2214	PB1–106–125F	CCTCCATACAGCCATGGAAC	PB1–2195–2214R	GAAATCAATTCGTGCGTCAA
PA	1–1668	PA-1–21F	AGCGAAAGCAGGTACTGATCC	PA-1646–1668R	CATGTCTCCTATCTCGAGGACAC
	565–2233	PA-565–587F	GAAATGGCCAGTAGGGGTCTATG	PA-2208–2233R	AGTAGAAACAAGGTACTTTTTTGGAC
	74–2169	PA-74–93F	CGGAAAAGGCAATGAAAGAA	PA-2150–2169R	CAGTGCATGTGTGAGGAAGG
HA†	1–1776(1779)‡	HA-1–21F-T	AGCAAAAGCAGGGGTTCAATC	HA-1754–1776R-T	AGTAGAAACAAGGGTGTTTTTAACTA
	1–1776	HA-1–21F-T	AGCAAAAGCAGGGGTTCAATC	HA-1754–1776R-C	AGTAGAAACAAGGGTGTTTTCAACTA
	1–1776	HA-1–21F-C	AGCAAAAGCAGGGGTCCAATC	HA-1754–1776R-T	AGTAGAAACAAGGGTGTTTTTAACTA
	96–793	HA-96–115F	ACCATGCAAACAACTCGCAC	HA-774–793R	GATTGCATCGTCCGGTTTTA
NP	1–1565	NP-1–24F	AGCAAAAGCAGGGTAGATAATCAC	NP-1541–1565R	AGTAGAAACAAGGGTATTTTTCTTTA ATTG
	51–1487	NP-51–70F	GTCTCAAGGCACCAAACGAT	NP-1468–1487R	ATGTCAAAGGAAGGCACGAT
NS	1–875	NS-1–23F	AGCAAAAGCAGGGTGACAAAAAC	NS-852–875R	AGTAGAAACAAGGGTGTTTTTTAT
	83–772	NS-83–102F	CAAACGATTTGCAGACCAAG	NS-752–772R	TCTGTTCGAAGCTGTTTTCTG

*All numbers listed refer to positive-sense orientation.

†To amplify the full-length HA segment, we used three different combinations of primers, based on the predetermined terminal sequences of the vRNA segments of each virus: HA-1–21F-T/HA-1754–1776R-T were used for UT3040I/II, UT31312I/II/III, UT31394I/II, and UT36250I/II; HA-1–21F-T/HA-1754–1776R-C were used for UT31413I/II; and HA-1–21F-C/HA-1754–1776R-T were used for UT36282I/II and UT36285I/II.

‡The HA genes of UT3040I and UT36250I are 1779 nt in length because of three additional nucleotides at the multibasic cleavage site.

Technical Appendix Table 2. Summary of nonsynonymous and synonymous single-nucleotide polymorphisms (SNPs) detected at ≥1%

		Viral RNA segment											
	Date of sample	PB2		PB1		PA		HA		NP		NS	
Virus isolate	collection	N*	S†	N	S	N	S	N	S	N	S	N	S
UT3040I	2004 Jan 6	8	2	6	10	10	0	1	4	1	3	1	0
UT3040II	2004 Jan 7	NA‡	NA	0	0	0	1	1	0	NA	NA	0	0
UT31312I	2007 Jul 25	3	2	0	1	0	0	1	3	1	0	0	0
UT31312II	2007 Jul 26	4	1	0	0	0	0	5	0	1	0	1	0
UT31312III	2007 Jul 25	4	0	0	0	0	0	6	2	0	1	0	0
UT31394I	2008 Jan 17	12	3	6	10	5	3	11	8	5	0	2	1
UT31394II	2008 Jan 17	6	1	3	2	4	0	4	3	4	0	1	0
UT31413I	2008 Feb 13	0	0	9	2	5	5	1	0	6	3	4	0
UT31413II	2008 Feb 13	0	1	0	3	0	1	5	8	0	0	2	0
UT36250I	2010 Mar 10	12	8	9	7	11	4	14	1	7	0	3	0
UT36250II	2010 Mar 11	0	0	NA	NA	NA	NA	6	2	NA	NA	1	0
UT36282I	2010 Apr 1	3	3	2	5	1	2	3	2	2	1	0	0
UT36282II	2010 Apr 3	2	2	1	3	2	2	5	7	3	3	8	4
UT36285I	2010 Apr 4	0	1	NA	NA	0	1	0	0	0	0	0	0
UT36285II	2010 Apr 8	3	2	NA	NA	3	2	NA	NA	1	0	0	0

*N, nonsynonymous single-nucleotide polymorphism; NA, not applicable because of low sequence coverage; S, synonymous single-nucleotide polymorphism.

Technical Appendix Table 3. Genetic variability among influenza A(H5N1) virus samples isolated from infected persons, northern Vietnam, 2004–2010

		Major variant in sample		Minor variant in sample		Consensus of top 25
Viral protein,						blastn hits of avian H5
aa position	Virus isolate	Amino acid	Percentage	Amino acid	Percentage	viruses†
PB2						
15	UT36250I	R	69.7	C	26.9	R
	UT36250II	C	100.0	R	0.0	R
**627**	UT3040I	K	92.6	E	7.4	E
	UT31312I	E	67.9	K	32.1	E
	UT31312II	E	72.1	K	27.9	E
	UT31312III	K	64.7	E	35.3	E
	UT31394I	E	50.5	K	49.5	E

Viral protein, aa position	Virus isolate	Major variant in sample		Minor variant in sample		Consensus of top 25 blastn hits of avian H5 viruses†
		Amino acid	Percentage	Amino acid	Percentage	
	UT31394II	K	51.7	E	48.3	E
	UT36250I	E	74.3	K	25.7	E
PB1						
123	UT36250I	T	95.0	A	5.0	T
213	UT36282I	N	92.3	D	7.7	N
	UT36282II	N	94.4	D	5.6	N
254	UT31394I	F	94.3	L	5.7	F
	UT31394II	F	93.2	L	6.8	F
538	UT31394II	D	84.9	G	15.1	D
598	UT36250I	L	68.8	P	31.2	L
PA						
85	UT3040I	T	95.2	M	4.8	T
	UT3040II	M	100.0	T	0.0	T
90	UT3040I	V	94.8	M	5.2	V
142	UT3040I	K	87.2	E	12.8	K
260	UT31413I	F	94.5	S	5.5	F
325	UT31413I	P	94.9	S	5.1	P
387	UT3040I	V	94.2	I	5.8	V
417	UT3040I	L	91.4	V	8.6	L
	UT3040II	V	100.0	L	0.0	L
651	UT31394I	A	89.0	S	11.0	A
	UT31394II	A	90.1	S	9.9	A
HA‡						
54(+1)§	UT31394I	E	66.5	D	33.5	D
	UT31394II	D	54.5	E	45.5	D
67	UT31394II	A	91.7	T	8.3	A
92	UT31312II	N	60.7	K	39.3	N
**138**	UT3040I	A	94.7	V	5.3	A
	UT36250I	A	72.0	V	28.0	A
	UT36250II	A	80.2	V	19.8	A
	UT36282I	A	93.9	V	6.1	A
	UT36282II	A	94.7	V	5.3	A
**186**	UT3040II	N	88.6	K	11.4	N

Viral protein, aa position	Virus isolate	Major variant in sample		Minor variant in sample		Consensus of top 25 blastn hits of avian H5 viruses†
		Amino acid	Percentage	Amino acid	Percentage	
	UT31312III	N	86.8	K	13.2	N
203	UT31312III	S	85.4	P	14.6	S
207	UT31394I	S	79.0	T	21.0	S
	UT31394II	S	52.8	T	47.2	S
226	UT36250II	Q	86.0	K	14.0	Q
456	UT31312III	M	54.9	R	45.1	R
486	UT31413II	Y	94.7	H	5.3	Y
511	UT31413I	T	90.4	I	9.6	T
NP						
77	UT31394I	R	92.3	K	7.7	R
	UT31394II	R	79.1	K	20.9	R
430	UT36282II	A	92.5	V	7.5	T[#]
454	UT31394I	E	91.2	G	8.8	E
	UT31394II	E	79.0	G	21.0	E
NS1						
124	UT36250I	I	85.8	M	14.2	I
	UT36250II	I	78.2	M	21.8	I

*Listed are single-nucleotide polymorphisms detected in 5%–50% of the sequence reads directly derived from viral RNAs (single-nucleotide polymorphisms detected in more than half of the sequence reads were considered the major variant); also listed is 1 variant (at position 85 of PA) that was found in <5% of the reads at the first sampling time, but increased substantially in frequency at the second sampling time. HA, hemagglutinin; NP, nucleocapsid; NS, nonstructural; PA, polymerase acidic; PB, polymerase basic. Boldface indicates amino acid changes detected in samples isolated from several patients.

†To compare the amino acid sequences of the human H5N1 virus isolates tested here with those of closely related avian H5 viruses, each human H5N1 viral gene sequence was first BLASTn searched with avian H5N1 viruses. Then, the consensus amino acids of the top 25 BLASTn hits were retrieved.

‡For HA, the numbers refer to the amino acid positions in mature H3 HA.

§After position 54, H5 HA proteins possess an additional amino acid not found in H3 HA proteins.

#For the top 5 BLASTn (https://www.fludb.org/brc/blast.spg?method=ShowCleanInputPage&decorator=influenza) hits (i.e., the most closely related viruses), the consensus amino acid at this position is A.

Technical Appendix Table 2. List of sialosides used for glycan arrays

Glycan #	Common Name	Structure
1	Galβ(1-4)GlcNAcβ-ethyl-NH2	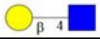
2	Galβ(1-4)GlcNAcβ(1-3)Galβ(1-3)GalNAcα-Thr-NH2	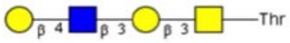
3	Galβ(1-4)GlcNAcβ(1-6)[Galβ(1-3)]-GalNAcα-Thr-NH2	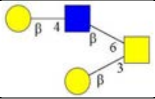
4	Galβ(1-4)GlcNAcβ(1-3)GalNAcα-Thr-NH2	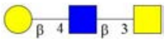
5	Galβ(1-4)GlcNAcβ(1-3)[Galβ(1-4)GlcNAcβ(1-6)]-GalNAcα-Thr-NH2	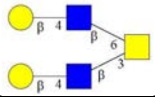
6	Galβ(1-4)GlcNAcβ(1-6)GalNAcα-Thr-NH2	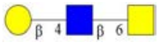
7	Galβ(1-4)GlcNAcβ(1-2)Manα (1-3)[Galβ(1-4)GlcNAcβ(1-2)Manα(1-6)]-Manβ(1-4)GlcNAcβ(1-4)GlcNAcβ-Asn-NH2	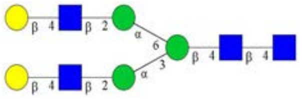
8	Galβ(1-4)GlcNAcβ(1-2)Manα(1-3)[Galβ(1-4)GlcNAcβ(1-2)Manα(1-6)]-Manβ(1-4)GlcNAcβ(1-4)[Fucα(1-6)]-GlcNAcβ-Asn-Ser-Thr-NH2	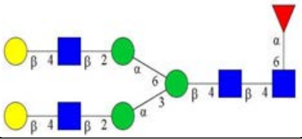
9	Galβ(1-4)GlcNAcβ(1-2)Manα(1-3){Galβ(1-4)GlcNAcβ(1-2)[Galβ(1-4)GlcNAcβ(1-2)]-Manα(1-6)}-Manβ(1-4)GlcNAcβ(1-4)GlcNAcβ-Asn-Lys-NH2	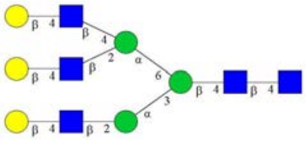
10	Galβ(1-4)GlcNAcβ(1-2)Manα(1-3){Galβ(1-4)GlcNAcβ(1-2)[Galβ(1-4)GlcNAcβ(1-2)]-Manα(1-6)}-Manβ(1-4)GlcNAcβ(1-4)[Fucα(1-6)]-GlcNAcβ-(Lys-Val-Ala)Asn-Lys-Thr-NH2	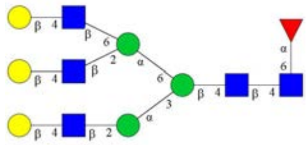
11	NeuAcα(2-3)Galβ(1-4)6-O-sulfo-GlcNAcβ-propyl-NH2	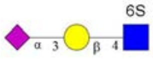
12	NeuAcα(2-3)Galβ(1-4)[Fucα(1-3)]-6-O-sulfo-GlcNAcβ-propyl-NH2	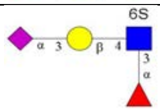
13	NeuAcα(2-3)6-O-sulfo-Galβ(1-4)GlcNAcβ-ethyl-NH2	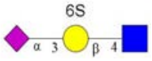
14	NeuAcα(2-3)6-O-sulfo-Galβ(1-4)[Fucα(1-3)]-GlcNAcβ-propyl-NH2	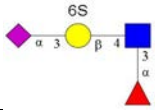
15	NeuAcα(2-3)Galβ(1-3)6-O-sulfo-GlcNAcβ-propyl-NH2	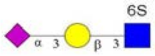
16	NeuAcα(2-3)Galβ(1-4)Glcβ-ethyl-NH2	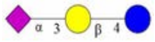
17	NeuAcα(2-3)Galβ(1-4)GlcNAcβ-ethyl-NH2	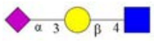
18	NeuAcα(2-3)Galβ(1-4)GlcNAcβ(1-3)Galβ(1-4)GlcNAcβ-ethyl-NH2	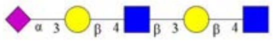
19	NeuAcα(2-3)Galβ(1-4)GlcNAcβ(1-3)Galβ(1-4)GlcNAcβ(1-3)Galβ(1-4)GlcNAcβ-ethyl-NH2	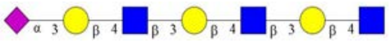

Glycan #	Common Name	Structure
**20**	NeuAcα(2-3)GalNAcβ(1-4)GlcNAcβ-ethyl-NH2	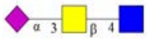
**21**	NeuAcα(2-3)Galβ(1-3)GlcNAcβ-ethyl-NH2	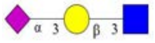
**22**	NeuAcα(2-3)Galβ(1-3)GlcNAcβ(1-3)Galβ(1-4)GlcNAcβ-ethyl-NH2	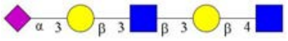
**23**	NeuAcα(2-3)Galβ(1-3)GlcNAcβ(1-3)Galβ(1-3)GlcNAcβ-ethyl-NH2	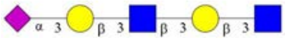
**24**	NeuAcα(2-3)Galβ(1-3)GalNAcβ(1-3)Galα(1-4)Galβ(1-4)Glcβ-ethyl-NH2	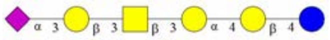
**25**	NeuAcα(2-3)Galβ(1-3)GalNAcα-Thr-NH2	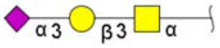
**26**	NeuAcα(2-3)Galβ(1-4)GlcNAcβ(1-3)Galβ(1-3)GalNAcα-Thr-NH2	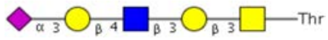
**27**	NeuAcα(2-3)Galβ(1-4)GlcNAcβ(1-3)Galβ(1-4)GlcNAcβ(1-3)Galβ(1-3)GalNAcα-Thr-NH2	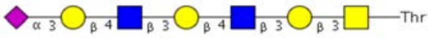
**28**	NeuAcα(2-3)Galβ(1-4)GlcNAcβ(1-3)Galβ(1-4)GlcNAcβ(1-3)Galβ(1-4)GlcNAcβ(1-3)Galβ(1-3)GalNAcα-Thr-NH2	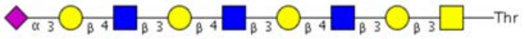
**29**	NeuAcα(2-3)Galβ(1-4)GlcNAcβ(1-3)Galβ(1-4)GlcNAcβ(1-3)Galβ(1-4)GlcNAcβ(1-3)Galβ(1-4)GlcNAcβ(1-3)Galβ(1-3)GalNAcα-Thr-NH2	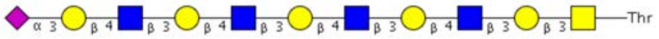
**30**	NeuAcα(2-3)Galβ(1-4)GlcNAcβ(1-3)Galβ(1-4)GlcNAcβ(1-3)Galβ(1-4)GlcNAcβ(1-3)Galβ(1-4)GlcNAcβ(1-3)Galβ(1-4)GlcNAcβ(1-3)Galβ(1-3)GalNAcα-Thr-NH2	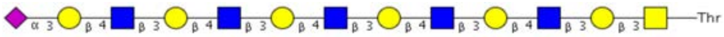
**31**	NeuAcα(2-3)Galβ(1-3)[GlcNAcβ(1-6)]-GalNAcα-Thr-NH2	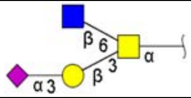
**32**	NeuAcα(2-3)Galβ(1-4)GlcNAcβ(1-6)[Galβ(1-3)]-GalNAcα-Thr-NH2	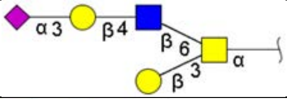
**33**	NeuAcα(2-3)Galβ(1-4)GlcNAcβ(1-3)Galβ(1-4)GlcNAcβ(1-6)[Galβ(1-3)]-GalNAcα-Thr-NH2	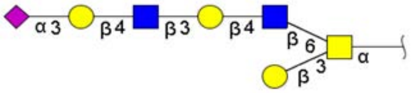
**34**	NeuAcα(2-3)Galβ(1-4)GlcNAcβ(1-3)Galβ(1-4)GlcNAcβ(1-3)Galβ(1-4)GlcNAcβ(1-6)[Galβ(1-3)]-GalNAcα-Thr-NH2	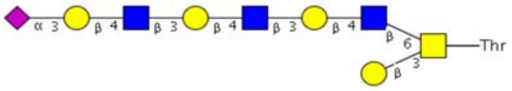
**35**	NeuAcα(2-3)Galβ(1-4)GlcNAcβ(1-3)Galβ(1-4)GlcNAcβ(1-3)Galβ(1-4)GlcNAcβ(1-3)Galβ(1-4)GlcNAcβ(1-6)[Galβ(1-3)]-GalNAcα-Thr-NH2	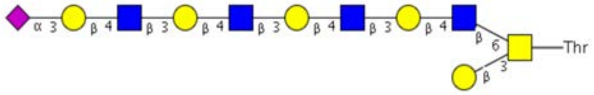
**36**	NeuAcα(2-3)Galβ(1-4)GlcNAcβ(1-3)Galβ(1-4)GlcNAcβ(1-3)Galβ(1-4)GlcNAcβ(1-3)Galβ(1-4)GlcNAcβ(1-3)Galβ(1-4)GlcNAcβ(1-6)[Galβ(1-3)]-GalNAcα-Thr-NH2	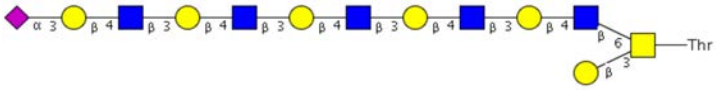

Glycan #	Common Name	Structure
**37**	NeuAcα(2-3)Galβ(1-4)GlcNAcβ(1-3)Galβ(1-4)GlcNAcβ(1-3)Galβ(1-4)GlcNAcβ(1-3)Galβ(1-4)GlcNAcβ(1-6)[NeuAcα(2-3)Galβ(1-4)GlcNAcβ(1-3)Galβ(1-4)GlcNAcβ(1-3)Galβ(1-4)GlcNAcβ(1-3)Galβ(1-3)]-GalNAcα-Thr-NH2	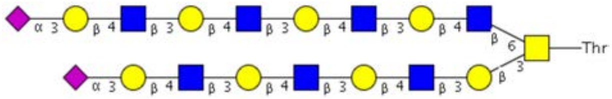
**38**	NeuAcα(2-3)Galβ(1-4)GlcNAcβ(1-3)Galβ(1-4)GlcNAcβ(1-3)Galβ(1-4)GlcNAcβ(1-3)Galβ(1-4)GlcNAcβ(1-3)Galβ(1-4)GlcNAcβ(1-6)[NeuAcα(2-3)Galβ(1-4)GlcNAcβ(1-3)Galβ(1-4)GlcNAcβ(1-3)Galβ(1-4)GlcNAcβ(1-3)Galβ(1-4)GlcNAcβ(1-3)Galβ(1-3)]-GalNAcα-Thr-NH2	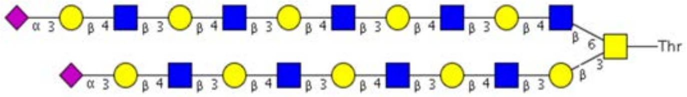
**39**	NeuAcα(2-3)Galβ(1-4)GlcNAcβ(1-3)GalNAcα-Thr-NH2	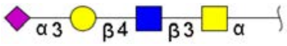
**40**	NeuAcα(2-3)Galβ(1-4)GlcNAcβ(1-3)Galβ(1-4)GlcNAcβ(1-3)GalNAcα-Thr-NH2	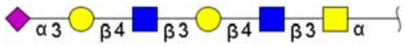
**41**	NeuAcα(2-3)Galβ(1-4)GlcNAcβ(1-3)Galβ(1-4)GlcNAcβ(1-3)Galβ(1-4)GlcNAcβ(1-3)GalNAcα-Thr-NH2	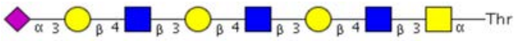
**42**	NeuAcα(2-3)Galβ(1-4)GlcNAcβ(1-3)Galβ(1-4)GlcNAcβ(1-3)Galβ(1-4)GlcNAcβ(1-3)Galβ(1-4)GlcNAcβ(1-3)GalNAcα-Thr-NH2	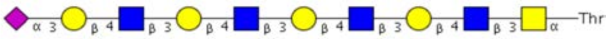
**43**	NeuAcα(2-3)Galβ(1-4)GlcNAcβ(1-3)Galβ(1-4)GlcNAcβ(1-3)Galβ(1-4)GlcNAcβ(1-3)Galβ(1-4)GlcNAcβ(1-3)Galβ(1-4)GlcNAcβ(1-3)GalNAcα-Thr-NH2	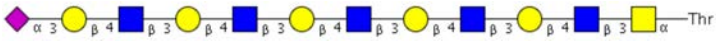
**44**	NeuAcα(2-3)Galβ(1-4)GlcNAcβ(1-3)[NeuAcα(2-3)Galβ(1-4)GlcNAcβ(1-6)]-GalNAcα-Thr-NH2	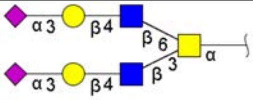
**45**	NeuAcα(2-3)Galβ(1-4)GlcNAcβ(1-3)Galβ(1-4)GlcNAcβ(1-3)[NeuAcα(2-3)Galβ(1-4)GlcNAcβ(1-3)Galβ(1-4)GlcNAcβ(1-6)]-GalNAcα-Thr-NH2	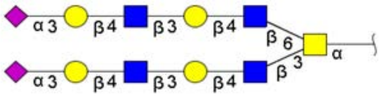
**46**	NeuAcα(2-3)Galβ(1-4)GlcNAcβ(1-3)Galβ(1-4)GlcNAcβ(1-3)Galβ(1-4)GlcNAcβ(1-3)[NeuAcα(2-3)Galβ(1-4)GlcNAcβ(1-3)Galβ(1-4)GlcNAcβ(1-3)Galβ(1-4)GlcNAcβ(1-6)]-GalNAcα-Thr-NH2	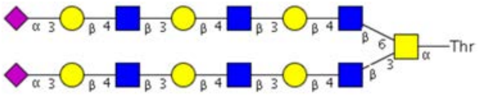
**47**	NeuAcα(2-3)Galβ(1-4)GlcNAcβ(1-3)Galβ(1-4)GlcNAcβ(1-3)Galβ(1-4)GlcNAcβ(1-3)Galβ(1-4)GlcNAcβ(1-3)[NeuAcα(2-3)Galβ(1-4)GlcNAcβ(1-3)Galβ(1-4)GlcNAcβ(1-3)Galβ(1-4)GlcNAcβ(1-3)Galβ(1-4)GlcNAcβ(1-6)]-GalNAcα-Thr-NH2	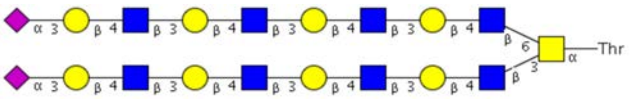

Glycan #	Common Name	Structure
**48**	NeuAcα(2-3)Galβ(1-4)GlcNAcβ(1-3)Galβ(1-4)GlcNAcβ(1-3)Galβ(1-4)GlcNAcβ(1-3)Galβ(1-4)GlcNAcβ(1-3)Galβ(1-4)GlcNAcβ(1-3)[NeuAcα(2-3)Galβ(1-4)GlcNAcβ(1-3)Galβ(1-4)GlcNAcβ(1-3)Galβ(1-4)GlcNAcβ(1-3)Galβ(1-4)GlcNAcβ(1-3)Galβ(1-4)GlcNAcβ(1-6)]-GalNAcα-Thr-NH2	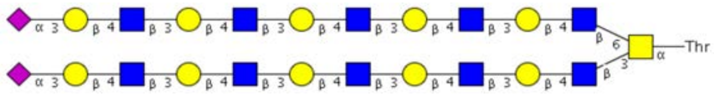
**49**	NeuAcα(2-3)Galβ(1-4)GlcNAcβ(1-3)Galβ(1-4)GlcNAcβ(1-3)Galβ(1-4)GlcNAcβ(1-3)Galβ(1-4)GlcNAcβ(1-6)GalNAcα-Thr-NH2	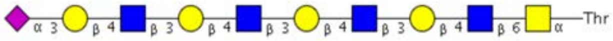
**50**	NeuAcα(2-3)Galβ(1-4)GlcNAcβ(1-3)Galβ(1-4)GlcNAcβ(1-3)Galβ(1-4)GlcNAcβ(1-3)Galβ(1-4)GlcNAcβ(1-3)Galβ(1-4)GlcNAcβ(1-6)GalNAcα-Thr-NH2	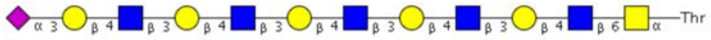
**51**	NeuAcα(2-3)Galβ(1-3)GlcNAcβ(1-3)Galβ(1-4)GlcNAcβ(1-6)[NeuAcα(2-3)Galβ(1-3)GlcNAcβ(1-3)] Galβ(1-4)GlcNAcβ-ethyl-NH2	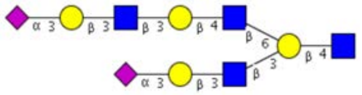
**52**	NeuAcα(2-3)Galβ(1-4)GlcNAcβ(1-3)Galβ(1-4)GlcNAcβ(1-3)Galβ(1-4)GlcNAcβ(1-6)[NeuAcα(2-3)Galβ(1-4)GlcNAcβ(1-3)Galβ(1-4)GlcNAcβ(1-3)] Galβ(1-4)GlcNAcβ-ethyl-NH2	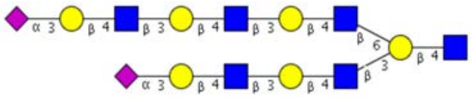
**53**	NeuAcα(2-3)Galβ(1-4)GlcNAcβ(1-2)Manα(1-3)[NeuAcα(2-3)Galβ(1-4)GlcNAcβ(1-2)Manα(1-6)]-Manβ(1-4)GlcNAcβ(1-4)GlcNAcβ-Asn-NH2	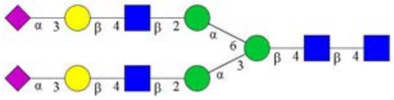
**54**	NeuAcα(2-3)Galβ(1-4)GlcNAcβ(1-3)Galβ(1-4)GlcNAcβ(1-2)Manα(1-3)[NeuAcα(2-3)Galβ(1-4)GlcNAcβ(1-3)Galβ(1-4)GlcNAcβ(1-2)Manα(1-6)]-Manβ(1-4)GlcNAcβ(1-4)GlcNAcβ-Asn-NH2	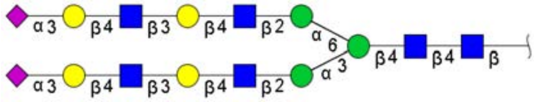
**55**	NeuAcα(2-3)Galβ(1-4)GlcNAcβ(1-3)Galβ(1-4)GlcNAcβ(1-3)Galβ(1-4)GlcNAcβ(1-2)Manα(1-3)[NeuAcα(2-3)Galβ(1-4)GlcNAcβ(1-3)Galβ(1-4)GlcNAcβ(1-3)Galβ(1-4)GlcNAcβ(1-2)Manα(1-6)]-Manβ(1-4)GlcNAcβ(1-4)GlcNAcβ-Asn-NH2	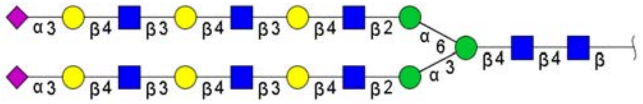
**56**	NeuAcα(2-3)Galβ(1-4)GlcNAcβ(1-3)Galβ(1-4)GlcNAcβ(1-2)Manα(1-3)[NeuAcα(2-3)Galβ(1-4)GlcNAcβ(1-3)Galβ(1-4)GlcNAcβ(1-2)Manα(1-6)]-Manβ(1-4)GlcNAcβ(1-4)GlcNAcβ-(Lys-Val-Ala)Asn-Lys-Thr-NH2	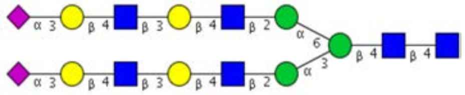
**57**	NeuAcα(2-3)Galβ(1-4)GlcNAcβ(1-3)Galβ(1-4)GlcNAcβ(1-3)Galβ(1-4)GlcNAcβ(1-2)Manα(1-3)[NeuAcα(2-3)Galβ(1-4)GlcNAcβ(1-3)Galβ(1-4)GlcNAcβ(1-3)Galβ(1-4)GlcNAcβ(1-2)Manα(1-6)]-Manβ(1-4)GlcNAcβ(1-4)GlcNAcβ-(Lys-Val-Ala)Asn-Lys-Thr-NH2	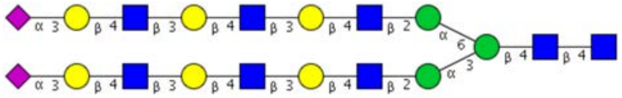

Glycan #	Common Name	Structure
58	NeuAcα(2-3)Galβ(1-4)GlcNAcβ(1-3)Galβ(1-4)GlcNAcβ(1-3)Galβ(1-4)GlcNAcβ(1-3)Galβ(1-4)GlcNAcβ(1-2)Manα(1-3)[NeuAcα(2-3)Galβ(1-4)GlcNAcβ(1-3)Galβ(1-4)GlcNAcβ(1-3)Galβ(1-4)GlcNAcβ(1-3)Galβ(1-4)GlcNAcβ(1-2)Manα(1-6)]-Manβ(1-4)GlcNAcβ(1-4)GlcNAcβ-(Lys-Val-Ala)Asn-Lys-Thr-NH2	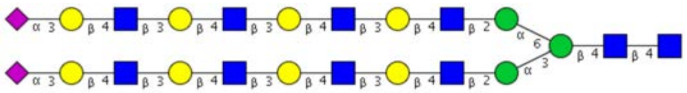
59	NeuAcα(2-3)Galβ(1-4)GlcNAcβ(1-3)Galβ(1-4)GlcNAcβ(1-3)Galβ(1-4)GlcNAcβ(1-3)Galβ(1-4)GlcNAcβ(1-3)Galβ(1-4)GlcNAcβ(1-2)Manα(1-3)[NeuAcα(2-3)Galβ(1-4)GlcNAcβ(1-3)Galβ(1-4)GlcNAcβ(1-3)Galβ(1-4)GlcNAcβ(1-3)Galβ(1-4)GlcNAcβ(1-3)Galβ(1-4)GlcNAcβ(1-2)Manα(1-6)]-Manβ(1-4)GlcNAcβ(1-4)GlcNAcβ-(Lys-Val-Ala)Asn-Lys-Thr-NH2	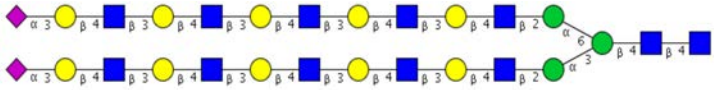
60	NeuAcα(2-3)Galβ(1-4)GlcNAcβ(1-3)Galβ(1-4)GlcNAcβ(1-3)Galβ(1-4)GlcNAcβ(1-2)Manα(1-3)[NeuAcα(2-3)Galβ(1-4)GlcNAcβ(1-3)Galβ(1-4)GlcNAcβ(1-3)Galβ(1-4)GlcNAcβ(1-2)Manα(1-6)]-Manβ(1-4)GlcNAcβ(1-4)[Fucα(1-6)]-GlcNAcβ-(Lys-Val-Ala)Asn-Lys-Thr-NH2	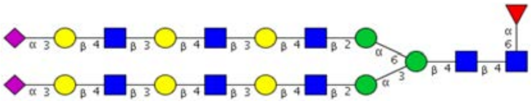
61	NeuAcα(2-3)Galβ(1-4)GlcNAcβ(1-3)Galβ(1-4)GlcNAcβ(1-3)Galβ(1-4)GlcNAcβ(1-3)Galβ(1-4)GlcNAcβ(1-2)Manα(1-3)[NeuAcα(2-3)Galβ(1-4)GlcNAcβ(1-3)Galβ(1-4)GlcNAcβ(1-3)Galβ(1-4)GlcNAcβ(1-3)Galβ(1-4)GlcNAcβ(1-2)Manα(1-6)]-Manβ(1-4)GlcNAcβ(1-4)[Fucα(1-6)]-GlcNAcβ-(Lys-Val-Ala)Asn-Lys-Thr-NH2	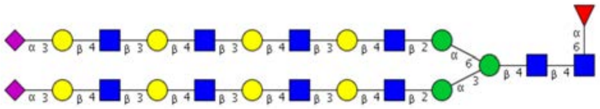
62	NeuAcα(2-3)Galβ(1-4)GlcNAcβ(1-3)Galβ(1-4)GlcNAcβ(1-2)Manα(1-3){NeuAcα(2-3)Galβ(1-4)GlcNAcβ(1-3)Galβ(1-4)GlcNAcβ(1-2)[NeuAcα(2-3)Galβ(1-4)GlcNAcβ(1-3)Galβ(1-4)GlcNAcβ(1-6)Manα(1-6)]}-Manβ(1-4)GlcNAcβ(1-4)GlcNAcβ-(Lys-Val-Ala)Asn-Lys-Thr-NH2	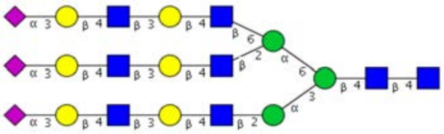
63	NeuAcα(2-3)Galβ(1-4)GlcNAcβ(1-3)Galβ(1-4)GlcNAcβ(1-3)Galβ(1-4)GlcNAcβ(1-2)Manα(1-3){NeuAcα(2-3)Galβ(1-4)GlcNAcβ(1-3)Galβ(1-4)GlcNAcβ(1-3)Galβ(1-4)GlcNAcβ(1-2)[NeuAcα(2-3)Galβ(1-4)GlcNAcβ(1-3)Galβ(1-4)GlcNAcβ(1-3)Galβ(1-4)GlcNAcβ(1-6)Manα(1-6)]}-Manβ(1-4)GlcNAcβ(1-4)GlcNAcβ-(Lys-Val-Ala)Asn-Lys-Thr-NH2	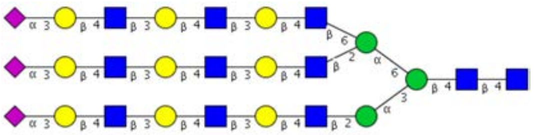

Glycan #	Common Name	Structure
64	NeuAcα(2-3)Galβ(1-4)GlcNAcβ(1-3)Galβ(1-4)GlcNAcβ(1-3)Galβ(1-4)GlcNAcβ(1-3)Galβ(1-4)GlcNAcβ(1-2)Manα(1-3){NeuAcα(2-3)Galβ(1-4)GlcNAcβ(1-3)Galβ(1-4)GlcNAcβ(1-3)Galβ(1-4)GlcNAcβ(1-3)Galβ(1-4)GlcNAcβ(1-2)[NeuAcα(2-3)Galβ(1-4)GlcNAcβ(1-3)Galβ(1-4)GlcNAcβ(1-3)Galβ(1-4)GlcNAcβ(1-3)Galβ(1-4)GlcNAcβ(1-6)Manα(1-6)]}-Manβ(1-4)GlcNAcβ(1-4)GlcNAcβ-(Lys-Val-Ala)Asn-Lys-Thr-NH2	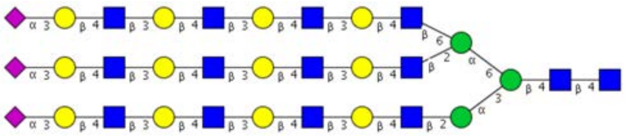
65	NeuAcα(2-3)Galβ(1-4)GlcNAcβ(1-3)Galβ(1-4)GlcNAcβ(1-2)Manα(1-3){NeuAcα(2-3)Galβ(1-4)GlcNAcβ(1-3)Galβ(1-4)GlcNAcβ(1-2)[NeuAcα(2-3)Galβ(1-4)GlcNAcβ(1-3)Galβ(1-4)GlcNAcβ(1-6)Manα(1-6)]}-Manβ(1-4)GlcNAcβ(1-4)[Fucα(1-6)]-GlcNAcβ-(Lys-Val-Ala)Asn-Lys-Thr-NH2	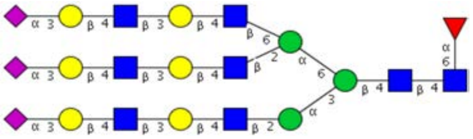
66	NeuAcα(2-3)Galβ(1-4)GlcNAcβ(1-3)Galβ(1-4)GlcNAcβ(1-3)Galβ(1-4)GlcNAcβ(1-2)Manα(1-3){NeuAcα(2-3)Galβ(1-4)GlcNAcβ(1-3)Galβ(1-4)GlcNAcβ(1-3)Galβ(1-4)GlcNAcβ(1-2)[NeuAcα(2-3)Galβ(1-4)GlcNAcβ(1-3)Galβ(1-4)GlcNAcβ(1-3)Galβ(1-4)GlcNAcβ(1-6)Manα(1-6)]}-Manβ(1-4)GlcNAcβ(1-4)[Fucα(1-6)]-GlcNAcβ-(Lys-Val-Ala)Asn-Lys-Thr-NH2	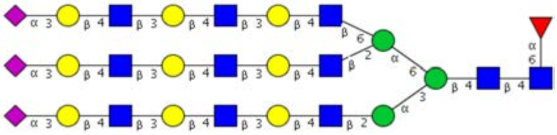
67	NeuAcα(2-3)Galβ(1-4)GlcNAcβ(1-3)Galβ(1-4)GlcNAcβ(1-3)Galβ(1-4)GlcNAcβ(1-3)Galβ(1-4)GlcNAcβ(1-2)Manα(1-3){NeuAcα(2-3)Galβ(1-4)GlcNAcβ(1-3)Galβ(1-4)GlcNAcβ(1-3)Galβ(1-4)GlcNAcβ(1-3)Galβ(1-4)GlcNAcβ(1-2)[NeuAcα(2-3)Galβ(1-4)GlcNAcβ(1-3)Galβ(1-4)GlcNAcβ(1-3)Galβ(1-4)GlcNAcβ(1-3)Galβ(1-4)GlcNAcβ(1-6)Manα(1-6)]}-Manβ(1-4)GlcNAcβ(1-4)[Fucα(1-6)]-GlcNAcβ-(Lys-Val-Ala)Asn-Lys-Thr-NH2	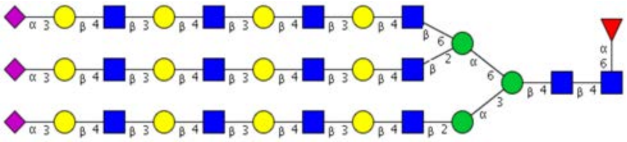
68	Gn/3'SLN/3'SLN-TriN	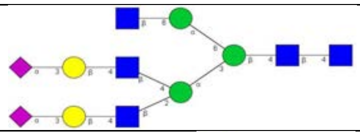
69	NeuAcα(2-3)[GalNAcβ(1-4)]-Galβ(1-4)GlcNAcβ-ethyl-NH2	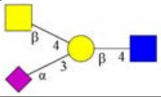
70	NeuAcα(2-3)[GalNAcβ(1-4)]-Galβ(1-4)Glcβ-ethyl-NH2	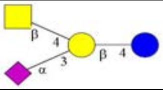
71	Galβ(1-3)GalNAcβ(1-4)[NeuAcα(2-3)]-Galβ(1-4)Glcβ-ethyl-NH2	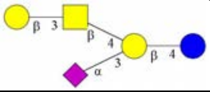

Glycan #	Common Name	Structure
72	NeuAcα(2-3)Galβ(1-4)[Fucα(1-3)]-GlcNAcβ-propyl-NH2	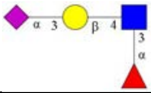
73	NeuAcα(2-3)Galβ(1-3)[Fucα(1-4)]-GlcNAcβ(1-3)Galβ(1-4)[Fucα(1-3)]-GlcNAcβ-ethyl-NH2	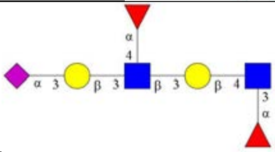
74	NeuAcα(2-3)Galβ(1-4)[Fucα(1-3)]-GlcNAcβ(1-3)Galβ(1-4)[Fucα(1-3)]-GlcNAcβ-ethyl-NH2	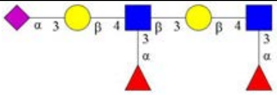
75	NeuAcα(2-3)Galβ(1-4)[Fucα(1-3)]-GlcNAcβ(1-3)Galβ(1-4)[Fucα(1-3)]-GlcNAcβ(1-3)Galβ(1-4)[Fucα(1-3)]-GlcNAcβ-ethyl-NH2	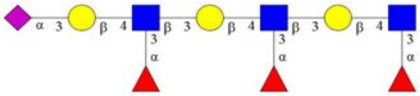
76	NeuAcα(2-3)Galβ(1-4)[Fucα(1-3)]-GlcNAcβ(1-3)Galβ(1-4)[Fucα(1-3)]-GlcNAcβ(1-3)Galβ(1-4)[Fucα(1-3)]-GlcNAcβ(1-3)Galβ(1-3)GalNAcα-Thr-NH2	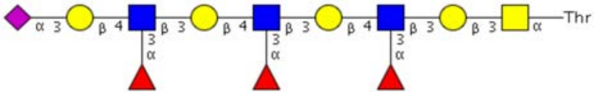
77	NeuAcα(2-3)Galβ(1-4)[Fucα(1-3)]-GlcNAcβ(1-3)Galβ(1-4)[Fucα(1-3)]-GlcNAcβ(1-3)Galβ(1-4)[Fucα(1-3)]-GlcNAcβ(1-3)GalNAcα-Thr-NH2	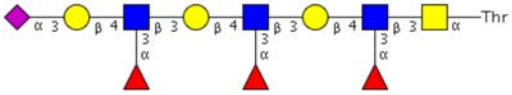
78	NeuAcα(2-3)Galβ(1-4)[Fucα(1-3)]-GlcNAcβ(1-3)Galβ(1-4)[Fucα(1-3)]-GlcNAcβ(1-3)Galβ(1-4)[Fucα(1-3)]-GlcNAcβ(1-3)[NeuAcα(2-3)Galβ(1-4)[Fucα(1-3)]-GlcNAcβ(1-3)Galβ(1-4)[Fucα(1-3)]-GlcNAcβ(1-3)Galβ(1-4)[Fucα(1-3)]-GlcNAcβ(1-6)]-GalNAcα-Thr-NH2	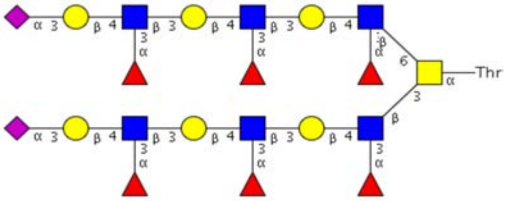
79	NeuAcα(2-3)Galβ(1-4)[Fucα(1-3)]-GlcNAcβ(1-2)Manα(1-3)[NeuAcα(2-3)Galβ(1-4)[Fucα(1-3)]-GlcNAcβ(1-2)Manα(1-6)]-Manβ(1-4)GlcNAcβ(1-4)GlcNAcβ-(Lys-Val-Ala)Asn-(Lys-Thr)NH2	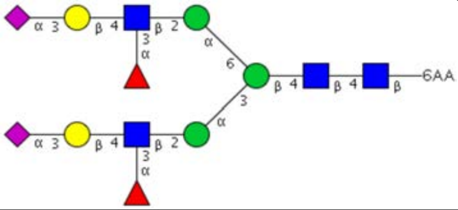
80	NeuAcα(2-6)Galb(1-4)(6S)GlcNacb-ethyl-NH2	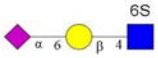
81	NeuAcα(2-6)Galβ(1-4)6-O-sulfo-GlcNAcβ-propyl-NH2	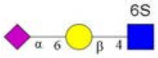
82	NeuAcα(2-6)Galβ(1-4)Glcβ-ethyl-NH2	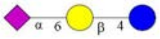
83	NeuAcα(2-6)Galβ(1-4)GlcNAcβ-ethyl-NH2	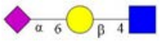
84	NeuAcα(2-6)Galβ(1-4)GlcNAcβ(1-3)Galβ(1-4)GlcNAcβ-ethyl-NH2	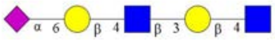
85	NeuAcα(2-6)Galβ(1-4)GlcNAcβ(1-3)Galβ(1-4)GlcNAcβ(1-3)Galβ(1-4)GlcNAcβ-ethyl-NH2	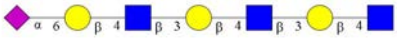
86	NeuAcα(2-6)GalNAcβ(1-4)GlcNAcβ-ethyl-NH2	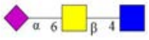
87	NeuAcα(2-6)Galβ(1-4)GlcNAcβ(1-3)Galβ(1-3)GalNAcα-Thr-NH2	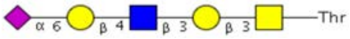

Glycan #	Common Name	Structure
88	NeuAcα(2-6)Galβ(1-4)GlcNAcβ(1-3)Galβ(1-4)GlcNAcβ(1-3)Galβ(1-3)GalNAcα-Thr-NH2	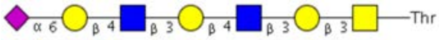
89	NeuAcα(2-6)Galβ(1-4)GlcNAcβ(1-3)Galβ(1-4)GlcNAcβ(1-3)Galβ(1-4)GlcNAcβ(1-3)Galβ(1-3)GalNAcα-Thr-NH2	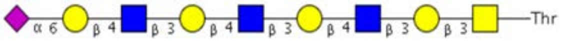
90	NeuAcα(2-6)Galβ(1-4)GlcNAcβ(1-3)Galβ(1-4)GlcNAcβ(1-3)Galβ(1-4)GlcNAcβ(1-3)Galβ(1-4)GlcNAcβ(1-3)Galβ(1-3)GalNAcα-Thr-NH2	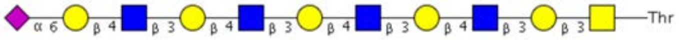
91	NeuAcα(2-6)Galβ(1-4)GlcNAcβ(1-3)Galβ(1-4)GlcNAcβ(1-3)Galβ(1-4)GlcNAcβ(1-3)Galβ(1-4)GlcNAcβ(1-3)Galβ(1-4)GlcNAcβ(1-3)Galβ(1-3)GalNAcα-Thr-NH2	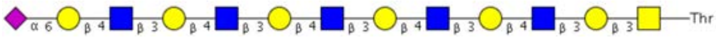
92	NeuAcα(2-6)Galβ(1-4)GlcNAcβ(1-6)[Galβ(1-3)]-GalNAcα-Thr-NH2	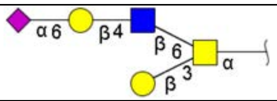
93	NeuAcα(2-6)Galβ(1-4)GlcNAcβ(1-3)Galβ(1-4)GlcNAcβ(1-6)[Galβ(1-3)]-GalNAcα-Thr-NH2	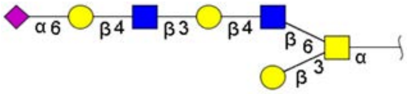
94	NeuAcα(2-6)Galβ(1-4)GlcNAcβ(1-3)Galβ(1-4)GlcNAcβ(1-3)Galβ(1-4)GlcNAcβ(1-6)[Galβ(1-3)]-GalNAcα-Thr-NH2	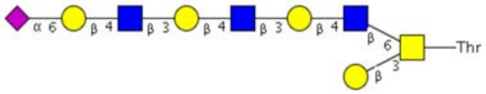
95	NeuAcα(2-6)Galβ(1-4)GlcNAcβ(1-3)Galβ(1-4)GlcNAcβ(1-3)Galβ(1-4)GlcNAcβ(1-3)Galβ(1-4)GlcNAcβ(1-6)[Galβ(1-3)]-GalNAcα-Thr-NH2	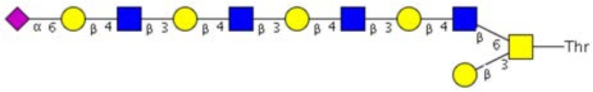
96	NeuAcα(2-6)Galβ(1-4)GlcNAcβ(1-3)Galβ(1-4)GlcNAcβ(1-3)Galβ(1-4)GlcNAcβ(1-3)Galβ(1-4)GlcNAcβ(1-3)Galβ(1-4)GlcNAcβ(1-6)[Galβ(1-3)]-GalNAcα-Thr-NH2	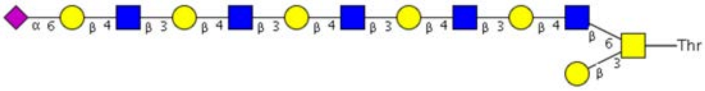
97	NeuAcα(2-6)Galβ(1-4)GlcNAcβ(1-3)Galβ(1-4)GlcNAcβ(1-3)Galβ(1-4)GlcNAcβ(1-3)Galβ(1-4)GlcNAcβ(1-6)[NeuAcα(2-6)Galβ(1-4)GlcNAcβ(1-3)Galβ(1-4)GlcNAcβ(1-3)Galβ(1-4)GlcNAcβ(1-3)Galβ(1-3)]-GalNAcα-Thr-NH2	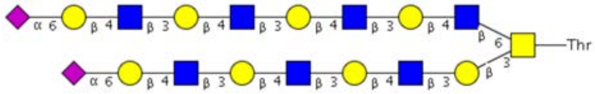
98	NeuAcα(2-6)Galβ(1-4)GlcNAcβ(1-3)Galβ(1-4)GlcNAcβ(1-3)Galβ(1-4)GlcNAcβ(1-3)Galβ(1-4)GlcNAcβ(1-3)Galβ(1-4)GlcNAcβ(1-6)[NeuAcα(2-6)Galβ(1-4)GlcNAcβ(1-3)Galβ(1-4)GlcNAcβ(1-3)Galβ(1-4)GlcNAcβ(1-3)Galβ(1-4)GlcNAcβ(1-3)Galβ(1-3)]-GalNAcα-Thr-NH2	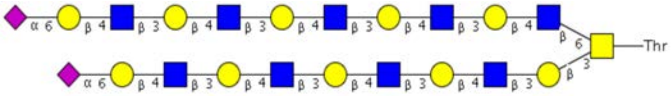
99	NeuAcα(2-6)Galβ(1-4)GlcNAcβ(1-3)GalNAcα-Thr-NH2	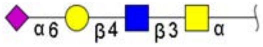
100	NeuAcα(2-6)Galβ(1-4)GlcNAcβ(1-3)Galβ(1-4)GlcNAcβ(1-3)GalNAcα-Thr-NH2	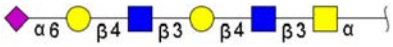
101	NeuAcα(2-6)Galβ(1-4)GlcNAcβ(1-3)Galβ(1-4)GlcNAcβ(1-3)Galβ(1-4)GlcNAcβ(1-3)GalNAcα-Thr-NH2	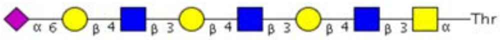

Glycan #	Common Name	Structure
**102**	NeuAcα(2-6)Galβ(1-4)GlcNAcβ(1-3)Galβ(1-4)GlcNAcβ(1-3)Galβ(1-4)GlcNAcβ(1-3)Galβ(1-4)GlcNAcβ(1-3)GalNAcα-Thr-NH2	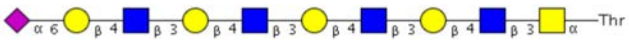
**103**	NeuAcα(2-6)Galβ(1-4)GlcNAcβ(1-3)Galβ(1-4)GlcNAcβ(1-3)Galβ(1-4)GlcNAcβ(1-3)Galβ(1-4)GlcNAcβ(1-3)Galβ(1-4)GlcNAcβ(1-3)GalNAcα-Thr-NH2	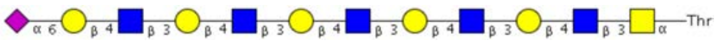
**104**	NeuAcα(2-6)Galβ(1-4)GlcNAcβ(1-3)[NeuAcα(2-6)Galβ(1-4)GlcNAcβ(1-6)]-GalNAcα-Thr-NH2	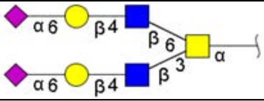
**105**	NeuAcα(2-6)Galβ(1-4)GlcNAcβ(1-3)Galβ(1-4)GlcNAcβ(1-3)[NeuAcα(2-6)Galβ(1-4)GlcNAcβ(1-3)Galβ(1-4)GlcNAcβ(1-6)]-GalNAcα-Thr-NH2	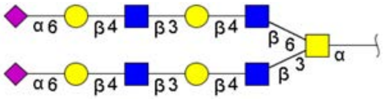
**106**	NeuAcα(2-6)Galβ(1-4)GlcNAcβ(1-3)Galβ(1-4)GlcNAcβ(1-3)Galβ(1-4)GlcNAcβ(1-3)[NeuAcα(2-6)Galβ(1-4)GlcNAcβ(1-3)Galβ(1-4)GlcNAcβ(1-3)Galβ(1-4)GlcNAcβ(1-6)]-GalNAcα-Thr-NH2	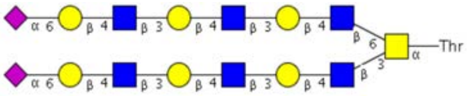
**107**	NeuAcα(2-6)Galβ(1-4)GlcNAcβ(1-3)Galβ(1-4)GlcNAcβ(1-3)Galβ(1-4)GlcNAcβ(1-3)Galβ(1-4)GlcNAcβ(1-3)[NeuAcα(2-6)Galβ(1-4)GlcNAcβ(1-3)Galβ(1-4)GlcNAcβ(1-3)Galβ(1-4)GlcNAcβ(1-3)Galβ(1-4)GlcNAcβ(1-6)]-GalNAcα-Thr-NH2	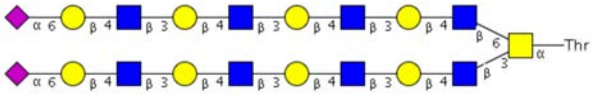
**108**	NeuAcα(2-6)Galβ(1-4)GlcNAcβ(1-3)Galβ(1-4)GlcNAcβ(1-3)Galβ(1-4)GlcNAcβ(1-3)Galβ(1-4)GlcNAcβ(1-3)Galβ(1-4)GlcNAcβ(1-3)[NeuAcα(2-6)Galβ(1-4)GlcNAcβ(1-3)Galβ(1-4)GlcNAcβ(1-3)Galβ(1-4)GlcNAcβ(1-3)Galβ(1-4)GlcNAcβ(1-3)Galβ(1-4)GlcNAcβ(1-6)]-GalNAcα-Thr-NH2	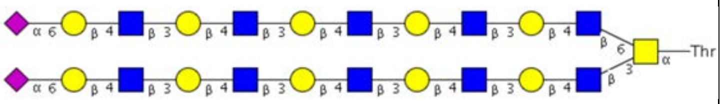
**109**	NeuAcα(2-6)Galβ(1-4)GlcNAcβ(1-3)Galβ(1-4)GlcNAcβ(1-3)Galβ(1-4)GlcNAcβ(1-3)Galβ(1-4)GlcNAcβ(1-6)GalNAcα-Thr-NH2	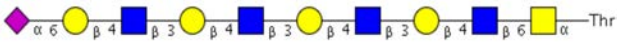
**110**	NeuAcα(2-6)Galβ(1-4)GlcNAcβ(1-3)Galβ(1-4)GlcNAcβ(1-3)Galβ(1-4)GlcNAcβ(1-3)Galβ(1-4)GlcNAcβ(1-3)Galβ(1-4)GlcNAcβ(1-6)GalNAcα-Thr-NH2	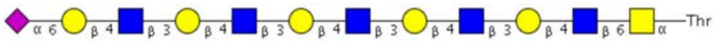
**111**	NeuAcα(2-6)Galβ(1-4)GlcNAcβ(1-3)Galβ(1-4)GlcNAcβ(1-3)Galβ(1-4)GlcNAcβ(1-6)[NeuAcα(2-6)Galβ(1-4)GlcNAcβ(1-3)Galβ(1-4)GlcNAcβ(1-3)] Galβ(1-4)GlcNAcβ-ethyl-NH2	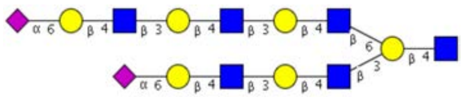
**112**	NeuAcα(2-6)Galβ(1-3)GlcNAcβ(1-3)Galβ(1-4)GlcNAcβ(1-6)[NeuAcα(2-6)Galβ(1-3)GlcNAcβ(1-3)] Galβ(1-4)GlcNAcβ-ethyl-NH2	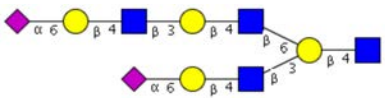

Glycan #	Common Name	Structure
113	Galβ(1-4)GlcNAcβ(1-2)Manα(1-3)[NeuAcα(2-6)Galβ(1-4)GlcNAcβ(1-2)Manα(1-6)]-Manβ(1-4)GlcNAcβ(1-4)GlcNAcβ-Asn-NH2	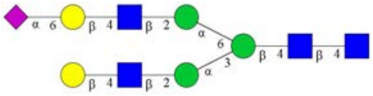
114	NeuAcα(2-6)Galβ(1-4)GlcNAcβ(1-2)Manα(1-3)[Galβ(1-4)GlcNAcβ(1-2)Manα(1-6)]-Manβ(1-4)GlcNAcβ(1-4)GlcNAcβ-Asn-NH2	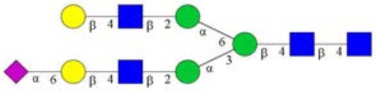
115	GlcNAcβ(1-2)Manα(1-3)[NeuAcα(2-6)Galβ(1-4)GlcNAcβ(1-2)Manα(1-6)]-Manβ(1-4)GlcNAcβ(1-4)GlcNAcβ-Asn-NH2	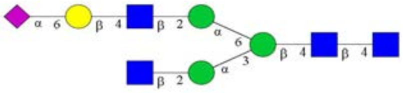
116	NeuAcα(2-6)Galβ(1-4)GlcNAcβ(1-2)Manα(1-3)[NeuAcα(2-6)Galβ(1-4)GlcNAcβ(1-2)Manα(1-6)]-Manβ(1-4)GlcNAcβ(1-4)GlcNAcβ-Asn-NH2	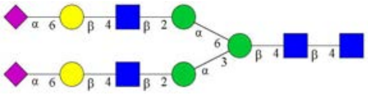
117	NeuAcα(2-6)Galβ(1-4)GlcNAcβ(1-3)Galβ(1-4)GlcNAcβ(1-2)Manα(1-3)[NeuAcα(2-6)Galβ(1-4)GlcNAcβ(1-3)Galβ(1-4)GlcNAcβ(1-2)Manα(1-6)]-Manβ(1-4)GlcNAcβ(1-4)GlcNAcβ-Asn-NH2	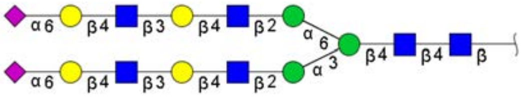
118	NeuAcα(2-6)Galβ(1-4)GlcNAcβ(1-3)Galβ(1-4)GlcNAcβ(1-2)Manα(1-3)[NeuAcα(2-6)Galβ(1-4)GlcNAcβ(1-3)Galβ(1-4)GlcNAcβ(1-2)Manα(1-6)]-Manβ(1-4)GlcNAcβ(1-4)GlcNAcβ-(Lys-Val-Ala)Asn-Lys-Thr-NH2	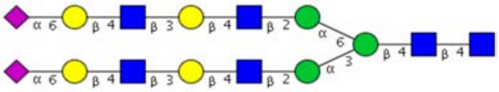
119	NeuAcα(2-6)Galβ(1-4)GlcNAcβ(1-3)Galβ(1-4)GlcNAcβ(1-3)Galβ(1-4)GlcNAcβ(1-2)Manα(1-3)[NeuAcα(2-6)Galβ(1-4)GlcNAcβ(1-3)Galβ(1-4)GlcNAcβ(1-3)Galβ(1-4)GlcNAcβ(1-2)Manα(1-6)]-Manβ(1-4)GlcNAcβ(1-4)GlcNAcβ-Asn-NH2	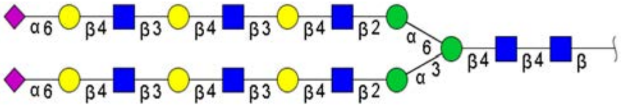
120	NeuAcα(2-6)Galβ(1-4)GlcNAcβ(1-3)Galβ(1-4)GlcNAcβ(1-3)Galβ(1-4)GlcNAcβ(1-2)Manα(1-3)[NeuAcα(2-6)Galβ(1-4)GlcNAcβ(1-3)Galβ(1-4)GlcNAcβ(1-3)Galβ(1-4)GlcNAcβ(1-2)Manα(1-6)]-Manβ(1-4)GlcNAcβ(1-4)GlcNAcβ-(Lys-Val-Ala)Asn-Lys-Thr-NH2	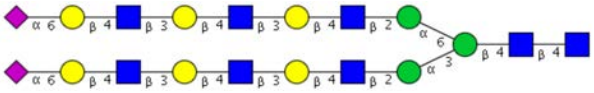
121	NeuAcα(2-6)Galβ(1-4)GlcNAcβ(1-3)Galβ(1-4)GlcNAcβ(1-3)Galβ(1-4)GlcNAcβ(1-3)Galβ(1-4)GlcNAcβ(1-2)Manα(1-3)[NeuAcα(2-6)Galβ(1-4)GlcNAcβ(1-3)Galβ(1-4)GlcNAcβ(1-3)Galβ(1-4)GlcNAcβ(1-3)Galβ(1-4)GlcNAcβ(1-2)Manα(1-6)]-Manβ(1-4)GlcNAcβ(1-4)GlcNAcβ-(Lys-Val-Ala)Asn-Lys-Thr-NH2	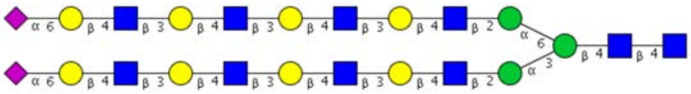

Glycan #	Common Name	Structure
122	NeuAcα(2-6)Galβ(1-4)GlcNAcβ(1-3)Galβ(1-4)GlcNAcβ(1-3)Galβ(1-4)GlcNAcβ(1-3)Galβ(1-4)GlcNAcβ(1-3)Galβ(1-4)GlcNAcβ(1-2)Manα(1-3)[NeuAcα(2-6)Galβ(1-4)GlcNAcβ(1-3)Galβ(1-4)GlcNAcβ(1-3)Galβ(1-4)GlcNAcβ(1-3)Galβ(1-4)GlcNAcβ(1-3)Galβ(1-4)GlcNAcβ(1-2)Manα(1-6)]-Manβ(1-4)GlcNAcβ(1-4)GlcNAcβ-(Lys-Val-Ala)Asn-Lys-Thr-NH2	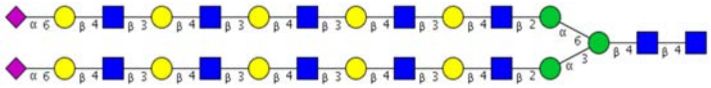
123	NeuAcα(2-6)Galβ(1-4)GlcNAcβ(1-3)Galβ(1-4)GlcNAcβ(1-2)Manα(1-3)[NeuAcα(2-6)Galβ(1-4)GlcNAcβ(1-3)Galβ(1-4)GlcNAcβ(1-2)Manα(1-6)]-Manβ(1-4)GlcNAcβ(1-4)[Fucα(1-6)]-GlcNAcβ-(Lys-Val-Ala)Asn-Lys-Thr-NH2	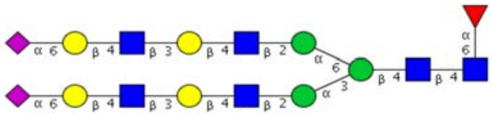
124	NeuAcα(2-6)Galβ(1-4)GlcNAcβ(1-3)Galβ(1-4)GlcNAcβ(1-3)Galβ(1-4)GlcNAcβ(1-2)Manα(1-3)[NeuAcα(2-6)Galβ(1-4)GlcNAcβ(1-3)Galβ(1-4)GlcNAcβ(1-3)Galβ(1-4)GlcNAcβ(1-2)Manα(1-6)]-Manβ(1-4)GlcNAcβ(1-4)[Fucα(1-6)]-GlcNAcβ-(Lys-Val-Ala)Asn-Lys-Thr-NH2	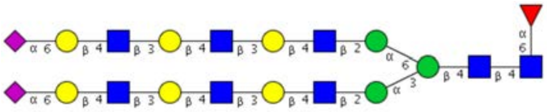
125	NeuAcα(2-6)Galβ(1-4)GlcNAcβ(1-3)Galβ(1-4)GlcNAcβ(1-3)Galβ(1-4)GlcNAcβ(1-3)Galβ(1-4)GlcNAcβ(1-2)Manα(1-3)[NeuAcα(2-6)Galβ(1-4)GlcNAcβ(1-3)Galβ(1-4)GlcNAcβ(1-3)Galβ(1-4)GlcNAcβ(1-3)Galβ(1-4)GlcNAcβ(1-2)Manα(1-6)]-Manβ(1-4)GlcNAcβ(1-4)[Fucα(1-6)]-GlcNAcβ-(Lys-Val-Ala)Asn-Lys-Thr-NH2	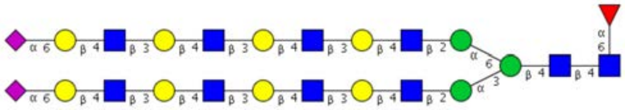
126	NeuAcα(2-6)Galβ(1-4)GlcNAcβ(1-3)Galβ(1-4)GlcNAcβ(1-2)Manα(1-3){NeuAcα(2-6)Galβ(1-4)GlcNAcβ(1-3)Galβ(1-4)GlcNAcβ(1-2)[NeuAcα(2-6)Galβ(1-4)GlcNAcβ(1-3)Galβ(1-4)GlcNAcβ(1-6)Manα(1-6)]}-Manβ(1-4)GlcNAcβ(1-4)GlcNAcβ-(Lys-Val-Ala)Asn-Lys-Thr-NH2	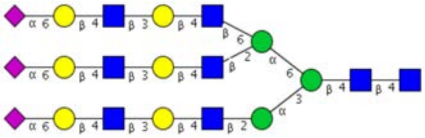
127	NeuAcα(2-6)Galβ(1-4)GlcNAcβ(1-3)Galβ(1-4)GlcNAcβ(1-3)Galβ(1-4)GlcNAcβ(1-2)Manα(1-3){NeuAcα(2-6)Galβ(1-4)GlcNAcβ(1-3)Galβ(1-4)GlcNAcβ(1-3)Galβ(1-4)GlcNAcβ(1-2)[NeuAcα(2-6)Galβ(1-4)GlcNAcβ(1-3)Galβ(1-4)GlcNAcβ(1-3)Galβ(1-4)GlcNAcβ(1-6)Manα(1-6)]}-Manβ(1-4)GlcNAcβ(1-4)GlcNAcβ-(Lys-Val-Ala)Asn-Lys-Thr-NH2	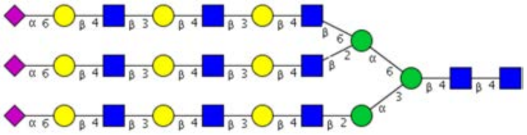

Glycan #	Common Name	Structure
**128**	NeuAcα(2-6)Galβ(1-4)GlcNAcβ(1-3)Galβ(1-4)GlcNAcβ(1-3)Galβ(1-4)GlcNAcβ(1-3)Galβ(1-4)GlcNAcβ(1-2)Manα(1-3){NeuAcα(2-6)Galβ(1-4)GlcNAcβ(1-3)Galβ(1-4)GlcNAcβ(1-3)Galβ(1-4)GlcNAcβ(1-3)Galβ(1-4)GlcNAcβ(1-2)[NeuAcα(2-6)Galβ(1-4)GlcNAcβ(1-3)Galβ(1-4)GlcNAcβ(1-3)Galβ(1-4)GlcNAcβ(1-3)Galβ(1-4)GlcNAcβ(1-6)Manα(1-6)]}-Manβ(1-4)GlcNAcβ(1-4)GlcNAcβ-(Lys-Val-Ala)Asn-Lys-Thr-NH2	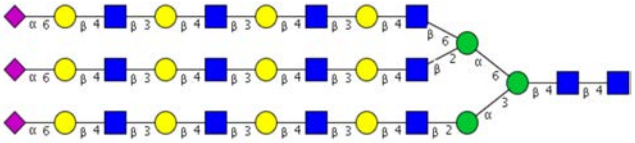
**129**	NeuAcα(2-6)Galβ(1-4)GlcNAcβ(1-3)Galβ(1-4)GlcNAcβ(1-2)Manα(1-3){NeuAcα(2-6)Galβ(1-4)GlcNAcβ(1-3)Galβ(1-4)GlcNAcβ(1-2)[NeuAcα(2-6)Galβ(1-4)GlcNAcβ(1-3)Galβ(1-4)GlcNAcβ(1-6)Manα(1-6)]}-Manβ(1-4)GlcNAcβ(1-4)[Fucα(1-6)]-GlcNAcβ-(Lys-Val-Ala)Asn-Lys-Thr-NH2	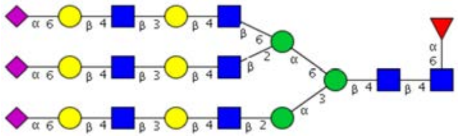
**130**	NeuAcα(2-6)Galβ(1-4)GlcNAcβ(1-3)Galβ(1-4)GlcNAcβ(1-3)Galβ(1-4)GlcNAcβ(1-2)Manα(1-3){NeuAcα(2-6)Galβ(1-4)GlcNAcβ(1-3)Galβ(1-4)GlcNAcβ(1-3)Galβ(1-4)GlcNAcβ(1-2)[NeuAcα(2-6)Galβ(1-4)GlcNAcβ(1-3)Galβ(1-4)GlcNAcβ(1-3)Galβ(1-4)GlcNAcβ(1-6)Manα(1-6)]}-Manβ(1-4)GlcNAcβ(1-4)[Fucα(1-6)]-GlcNAcβ-(Lys-Val-Ala)Asn-Lys-Thr-NH2	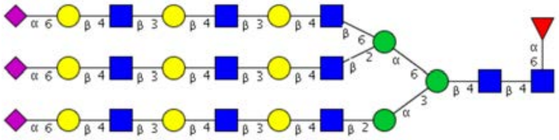
**131**	NeuAcα(2-6)Galβ(1-4)GlcNAcβ(1-3)Galβ(1-4)GlcNAcβ(1-3)Galβ(1-4)GlcNAcβ(1-3)Galβ(1-4)GlcNAcβ(1-2)Manα(1-3){NeuAcα(2-6)Galβ(1-4)GlcNAcβ(1-3)Galβ(1-4)GlcNAcβ(1-3)Galβ(1-4)GlcNAcβ(1-3)Galβ(1-4)GlcNAcβ(1-2)[NeuAcα(2-6)Galβ(1-4)GlcNAcβ(1-3)Galβ(1-4)GlcNAcβ(1-3)Galβ(1-4)GlcNAcβ(1-3)Galβ(1-4)GlcNAcβ(1-6)Manα(1-6)]}-Manβ(1-4)GlcNAcβ(1-4)[Fucα(1-6)]-GlcNAcβ-(Lys-Val-Ala)Asn-Lys-Thr-NH2	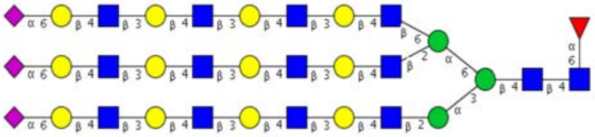
**132**	LN/6'SLN/6'SLN-TriN	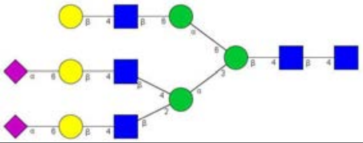
**133**	6'SLNLN/6'SLN/6'SLN-TriN	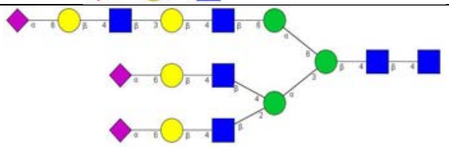

Glycan #	Common Name	Structure
**134**	6'SLN/LeX/LeX-TriN	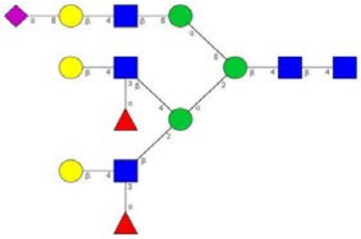
**135**	6'SLNLN/LeX/LeX-TriN	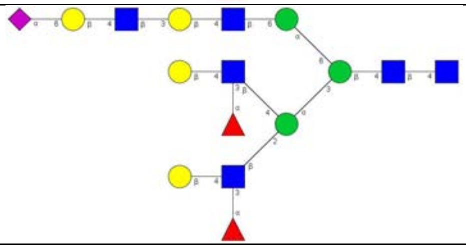

Technical Appendix Figure 1. List of sialosides used for glycan arrays. The first column indicating the glycan number is colored as follows: gray, glycan without sialic acid; blue, glycan with sialic acid in an α2,3-linkage; red, glycan with sialic acid in an α2,6-linkage.

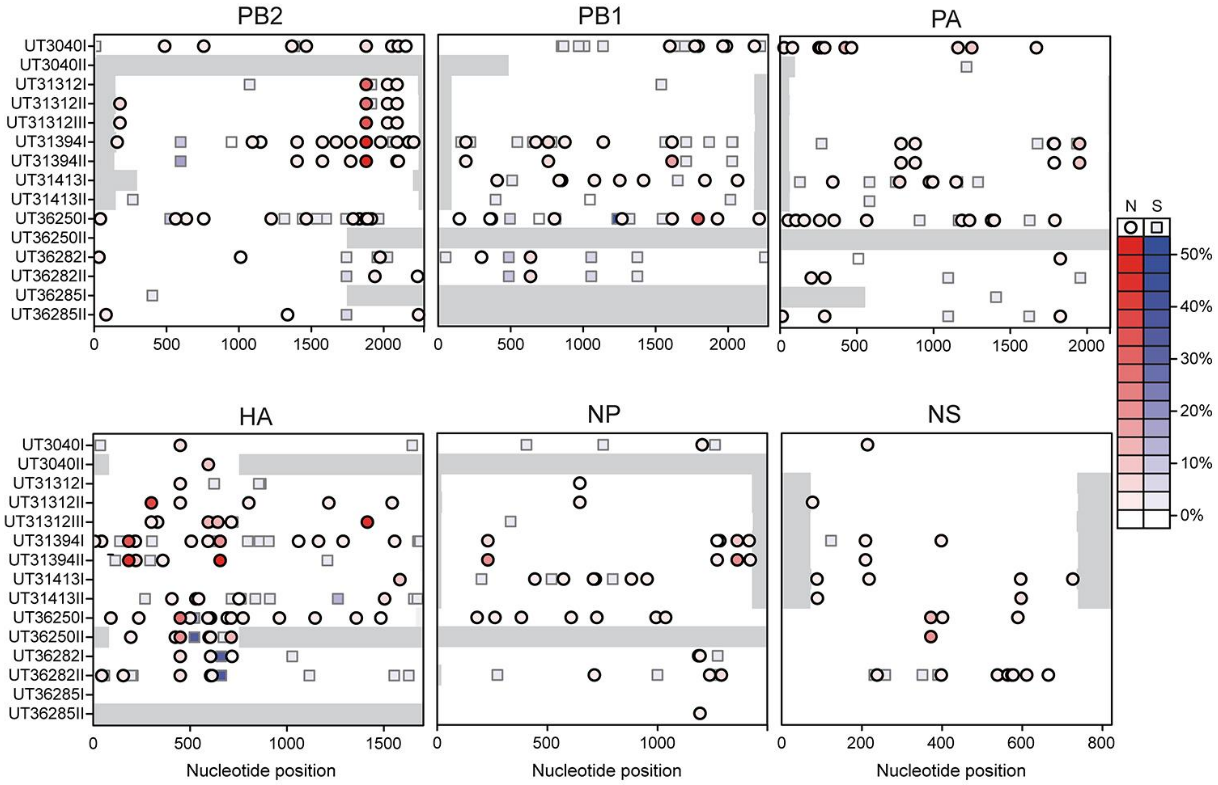

Technical Appendix Figure 2. Deep-sequencing reveals H5N1 influenza sequence variation among humans infected with H5N1 viruses. The PB2, PB1, PA, HA, NP, and NS vRNAs were amplified from throat swabs or tracheal aspirates and deep-sequenced. Single-nucleotide polymorphisms (SNPs) detected in ≥1% of sequence reads were identified by comparing sequence reads with the consensus sequence assembled for each sample. Grey bars indicate gene regions with insufficient sequence coverage (<100 sequence reads) to call variants. Squares and circles indicate synonymous and nonsynonymous SNPs, respectively. SNP frequencies are represented as a heatmap with synonymous and nonsynonymous mutations represented in blue and red, respectively. Since SNPs were called relative to their consensus nucleotide, the maximum frequency of each SNP is <50%.

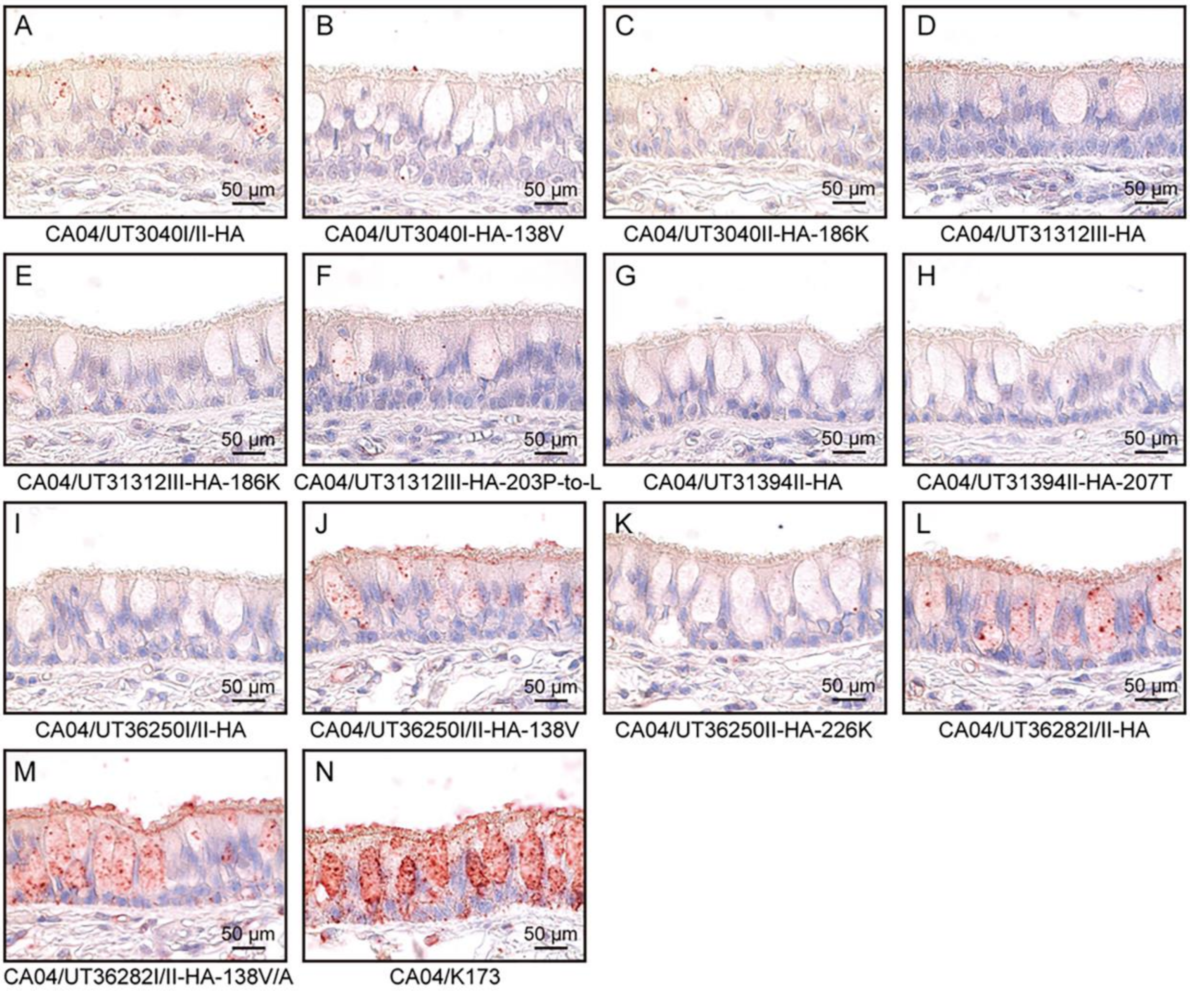

Technical Appendix Figure 3. Effect of amino acid variations in HA on tissue binding ability. Binding of viruses to human trachea sections, on which α2,6-linked sialic acids are present, was assessed. If two isolate numbers are listed (such as CA04/UT3040I/II), we tested the major sequence variant, which is identical between the two samples. FITC-labeled viruses were incubated with deparaffinized tissue sections and virus-tissue binding was detected by means of immunohistochemistry. Signals were observed as a dark-red color; images were obtained by using the upright microscope, Axio Imager.A2 (Zeiss, Oberkochen, Germany) with a 100 X objective lens. Nuclei were counterstained with Mayer's hematoxylin (Sigma Aldrich, St. Louis, MO, USA). CA04/K173 virus was used as a control.

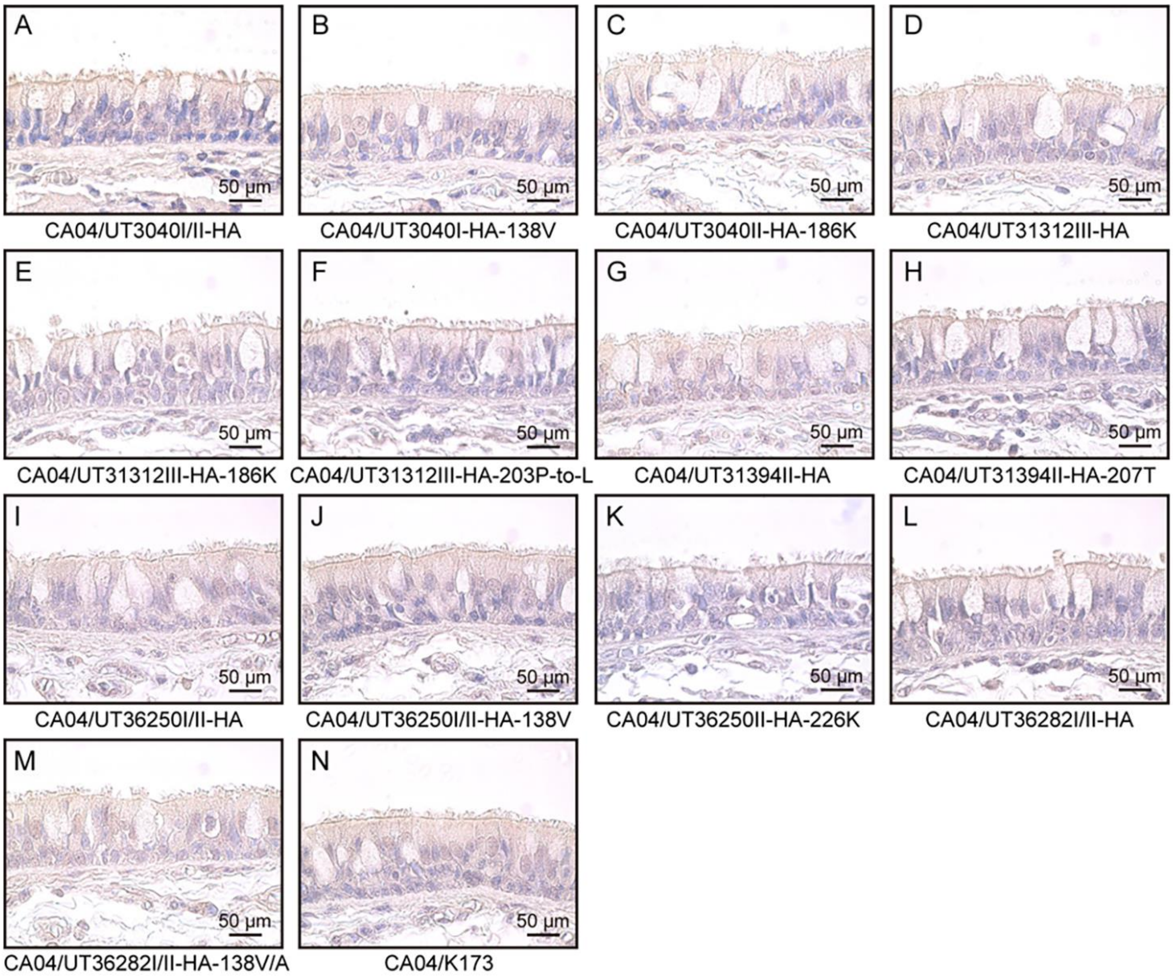

Technical Appendix Figure 4. Confirmation of virus binding specificity to sialic acids. To confirm virus binding specificity to sialic acids, we pretreated tracheal tissues with α(2→3,6,8,9) neuraminidase from *Arthrobacter ureafaciens* (Sigma Aldrich), which removes terminal sialic acids. Binding of viruses to the pretreated sections was assessed. If two isolate numbers are listed (such as CA04/UT3040I/II), we tested the major sequence variant, which is identical between the two samples. FITC-labeled viruses were incubated with the pretreated tissue sections and virus-tissue binding was detected by means of immunohistochemistry. Nuclei were counterstained with Mayer's hematoxylin (Sigma Aldrich).

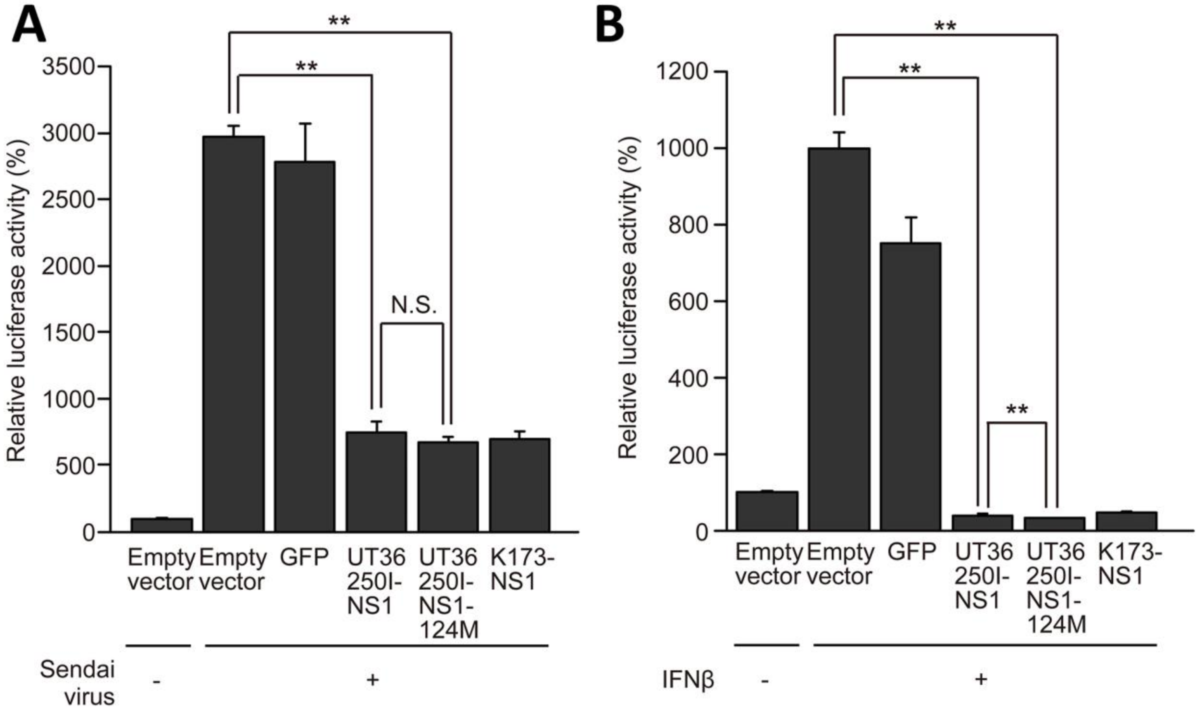

Technical Appendix Figure 5. Effect of an amino acid variation in NS1 on interferon antagonistic properties. (A) Effect of NS1 on interferon production. 293T cells were transfected with a plasmid encoding the wild-type or mutant NS gene and with a plasmid encoding the firefly luciferase gene under the control of the interferonβ promoter. An empty vector and a plasmid expressing GPF served as negative controls. A plasmid expressing the human influenza A virus K173 NS gene was used as a positive control. Twenty-four hours after transfection, cells were infected with 10^6 fluorescence-forming units of Sendai virus to stimulate interferon synthesis. The ability of NS1 to interfere with interferon production was measured 48 h later. (B) Effect of NS1 on interferon signaling. Cells were transfected with control plasmids or plasmids encoding the wild-type or mutant NS gene, and with a plasmid expressing luciferase under the control of an interferon-regulated stimulatory element (ISRE). The cells were incubated for 24 h and then treated with 100 units of human interferonβ for 6 h. In both assays, firefly luciferase activities were measured by using Luciferase Assay Reagent II (Promega). The experiments (each in triplicate) were repeated twice independently. Mean relative interferon antagonistic activities with standard deviations are shown. The activity of cells transfected with the pCAGGS empty vector (nontreated with Sendai virus or nonincubated with interferonβ) was set to 100%. The asterisks indicate a significant difference in interferon antagonistic activities between the ‘empty’ vector, UT36250I-NS1, and/or UT36250I-NS1–124M (**$p < 0.01$; one-way ANOVA followed by Tukey's post-hoc test; N.S.: not significant).
